# Generation of a platform strain for ionic liquid tolerance using adaptive laboratory evolution

**DOI:** 10.1186/s12934-017-0819-1

**Published:** 2017-11-16

**Authors:** Elsayed T. Mohamed, Shizeng Wang, Rebecca M. Lennen, Markus J. Herrgård, Blake A. Simmons, Steven W. Singer, Adam M. Feist

**Affiliations:** 1 Novo Nordisk Foundation Center for Biosustainability, Technical University of Denmark, Building 220, Kemitorvet, 2800 Kgs. Lyngby, Denmark; 2 Department of Bioengineering, University of California, 9500 Gilman Drive La Jolla, San Diego, CA 92093 USA; 3 Joint Bioenergy Institute, Emeryville, CA USA; 4 Biological Systems and Engineering Division, Lawrence Berkeley National Laboratory, Berkeley, CA USA; 5 State Key Laboratory of Chemical Resource Engineering, College of Life Science and Technology, Beijing University of Chemical Technology, Beijing, 100029 People’s Republic of China

**Keywords:** *Escherichia coli*, Renewable feedstocks, Ionic liquids, Adaptive laboratory evolution

## Abstract

**Background:**

There is a need to replace petroleum-derived with sustainable feedstocks for chemical production. Certain biomass feedstocks can meet this need as abundant, diverse, and renewable resources. Specific ionic liquids (ILs) can play a role in this process as promising candidates for chemical pretreatment and deconstruction of plant-based biomass feedstocks as they efficiently release carbohydrates which can be fermented. However, the most efficient pretreatment ILs are highly toxic to biological systems, such as microbial fermentations, and hinder subsequent bioprocessing of fermentative sugars obtained from IL-treated biomass.

**Methods:**

To generate strains capable of tolerating residual ILs present in treated feedstocks, a tolerance adaptive laboratory evolution (TALE) approach was developed and utilized to improve growth of two different *Escherichia coli* strains, DH1 and K-12 MG1655, in the presence of two different ionic liquids, 1-ethyl-3-methylimidazolium acetate ([C_2_C_1_Im][OAc]) and 1-butyl-3-methylimidazolium chloride ([C_4_C_1_Im]Cl). For multiple parallel replicate populations of *E. coli*, cells were repeatedly passed to select for improved fitness over the course of approximately 40 days. Clonal isolates were screened and the best performing isolates were subjected to whole genome sequencing.

**Results:**

The most prevalent mutations in tolerant clones occurred in transport processes related to the functions of *mdtJI*, a multidrug efflux pump, and *yhdP*, an uncharacterized transporter. Additional mutations were enriched in processes such as transcriptional regulation and nucleotide biosynthesis. Finally, the best-performing strains were compared to previously characterized tolerant strains and showed superior performance in tolerance of different IL and media combinations (i.e., cross tolerance) with robust growth at 8.5% (w/v) and detectable growth up to 11.9% (w/v) [C_2_C_1_Im][OAc].

**Conclusion:**

The generated strains thus represent the best performing platform strains available for bioproduction utilizing IL-treated renewable substrates, and the TALE method was highly successful in overcoming the general issue of substrate toxicity and has great promise for use in tolerance engineering.

**Electronic supplementary material:**

The online version of this article (10.1186/s12934-017-0819-1) contains supplementary material, which is available to authorized users.

## Background

There is a need to replace chemical and fuel production from fossil feedstocks with carbon neutral sources to retain the natural cycle of carbon emission and assimilation. Certain biomass feedstocks can play a major part in this need as they are abundant, diverse, and renewable. These biomass feedstocks include general plant-based materials like energy crops, crop residues, wood, wood residues, and grasses. Most of these materials have intrinsic value alongside with the added possibility of use as biomaterials [1]. Biomass feedstocks, however, possess a low energy density requiring a greater quantity of them to meet market demands [2]. Therefore, innovative approaches are necessary to make biomass feedstocks viable carbon sources to replace fossil feedstocks.

Lignocellulosic biomass can serve as a carbon neutral and abundant feedstock for bioprocesses [3]. In order to utilize lignocellulosic biomass for biochemical conversion in biorefineries, a pretreatment process is needed to remove the physical and chemical barriers to fully utilize the sugar substrates. The main aim of pretreatment is to increase the accessibility of cellulose, which then can be subjected to enzymatic saccharification to release fermentable sugars. This can be achieved through dissolution of hemicellulose and/or lignin, which coat the surface of cellulose [4]. There are several approaches for pretreatment of lignocellulosic biomass which include physical and chemical methods, but one of the most effective approaches is to directly release monomeric sugars through treatment with Ionic liquids (ILs) [5]. ILs are effective solvents for deconstruction and result in generating sugar feedstocks without significant loss of sugars due to degradation [4, 6, 7].

Despite its efficacy, IL pretreatment has some limitations, as some amounts of ILs remain from the pretreatment and these are often highly toxic to microbes used in downstream fermentation processes. Typically, a deconstruction hydrolysate has around 0.2–5% (w/v) of ionic liquid after the pretreatment [8]. One practice to overcome toxicity is to wash the pretreatment several times mostly with water, but this process adds significant purification costs [6]. Thus, an alternate approach to deal with toxicity by developing microbial platform strains that can tolerate residual ILs is needed. Limited tolerance toward ILs has been previously achieved using a range of techniques including rational design [8–10] and adaptive laboratory evolution (ALE) [11]. The rationally-designed strains generally introduced a non-native efflux pump for ILs which exerts a metabolic burden on the cells and a need for tight expression control. The previous ALE study showed promise for the approach [11], but the scope was limited to one IL, utilized a rich undefined media, and no genetic basis for the improved performance was presented. Nonetheless, this preliminary work revealed an opportunity to apply ALE for IL tolerance and can be used for comparison.

In the present work, the problem of IL toxicity is addressed using a systematic ALE strategy, Tolerance adaptive laboratory evolution (TALE), to generate *Escherichia coli* (*E. coli*) strains that were highly tolerant to the presence of ILs. The TALE method differs from previous efforts in that dynamic control is used to increase the amount of stress applied to cells to keep a strong selection pressure without crashing cultures due to overstressed growth conditions. The TALE approach for IL tolerance employed in the present study has two major advantages compared to manual ALE work (e.g., [11]). First, the TALE approach significantly improved fitness and final cell density in higher IL concentrations than the manual ALE approach. Additionally, the IL cross-tolerance phenotype exhibited by the best performing strains can expand the application of TALE-derived strains. Finally, these results were obtained over a significantly shorter time frame (40 vs 90 days) using an automated platform for performing TALE (details of the improvement are provided below).

In this study, two biotechnologically-relevant strains of *E. coli* (K-12 MG1655 and DH1) were exposed to increasing concentrations of two ILs; 1-butyl-3-methylimidazolium chloride and 1-ethyl-3-methylimidazolium acetate. Both of these targeted ILs are promising solvents for biomass pretreatment and were considered as a good candidates for IL-pretreated biomass [5, 7]. The exposure was performed over repeated exponential batch growth in parallel biological replicates. The evolved populations were screened and individual isolates were re-sequenced to identify key causal mutations. Selected isolates were compared against rationally-designed strains previously demonstrated to possess IL tolerance [8, 12]. The best performing strains showed markedly improved tolerance toward higher concentrations of ILs over rationally designed strains. The key mutations identified in this study provide a linkage between the IL tolerance phenotype and genotype.

## Methods

### Strains, reagents and equipment

Two *E. coli* strains were utilized: DH1 (ATCC 33849) and K-12 MG1655 (ATCC 47076). Two ionic liquids were utilized. 1-Butyl-3-methylimidazolium chloride ([C_4_C_1_Im]Cl) was purchased from Sigma-Aldrich (Basionics ST 70, BASF), and 1-ethyl-3-methylimidazolium acetate, ([C_2_C_1_Im][OAc]), was purchased from IOLITEC ionic Liquids Technologies GmbH (Heilbronn, Germany). Chemicals and components of the medium used for selecting the best performing strains were purchased from Sigma-Aldrich (St. Louis, USA) or VWR (West Chester, USA) unless otherwise noted.

M9 glucose medium contained 2 g/L glucose, 1× M9 salts, 2 mM MgSO_4_, 100 µM CaCl_2_ and 1× trace elements and Wolfe’s vitamin solution. Composition of 10× M9 salts solution consisted of 68 g/L Na_2_HPO_4_ anhydrous, 30 g/L KH_2_PO_4_, 5 g/L NaCl, and 10 g/L NH_4_Cl dissolved in Milli-Q filtered water and autoclaved. M9 trace elements was a 2000× solution containing of 3.0 g/L FeSO_4_·7H_2_O, 4.5 g/L ZnSO_4_·7H_2_O, 0.3 g/L CoCl_2_·6H_2_O, 0.4 g/L Na_2_MoO_4_·2H_2_O, 4.5 g/L CaCl_2_·H_2_O, 0.2 g/L CuSO_4_·2H_2_O, 1.0 g/L H_3_BO_3_, 15 g/L disodium ethylene-diamine-tetra-acetate, 0.1 g/L KI, 0.7 g/L MnCl_2_·4H_2_O and concentrated HCl dissolved in Milli-Q filtered water and sterile filtered. The final concentrations of the vitamin mix and trace elements in the M9 medium were 1×.

### Screening for tolerance in wild type strains

The two *E. coli* strains, DH1 and K-12 MG1655, were initially screened for their tolerance towards different concentrations of each IL in order to choose the starting concentration where the growth rate and final optical density were higher. A description of tolerance screening and tolerance phenotype in wild type strains (Additional file 2: Table S1). Cells from an overnight culture in LB medium were inoculated into cylindrical tubes containing 15 mL M9 glucose supplemented with varying concentrations of each ionic liquid. Inoculated tubes were temperature-controlled at 37 °C and fully aerated. Growth rates and final optical density were determined from 600 nm wavelength (OD_600_) measurements on a sunrise plate reader (Tecan, Männedorf, Switzerland).

### Adaptive laboratory evolution of IL tolerance

The bacterial cells were adaptively evolved under batch fermentation in M9 glucose supplemented with the initial ionic liquid concentration listed in Table 1, with increasing concentration of ILs applied over the course of the ALEs. Cells were serially passaged during exponential growth for approximately 40 days using an automated liquid-handler platform [13]. Pre-cultures for inoculating the starting culture were grown in M9 glucose and 150 µL of each pre-culture was used to inoculate each independent replicate with a working volume of 15 mL. Cells were cultured at 37 °C. OD_600_ was measured at a time determined algorithmically and once OD_600_ reached approximately OD_600_ 0.3, 150 µL was passed into a new tube with a fresh media containing ILs and a total working volume of 15 mL (i.e., a 1:100 ratio). The commonly experienced exponential growth phase was from time of inoculation to approximately OD_600_ 0.3 and the maximum final OD_600_ was approximately 0.4, thus the cells were passed during the exponential phase. The OD_600_ was measured by a Sunrise Plate Reader (Tecan Inc., Switzerland) and the common ratio between the plate reader OD_600_ and a benchtop spectrophotometer with a 1 cm path length is 4.2. Growth rates were determined by calculating the slope of the semi-log plot of log OD versus time using linear regression with the Polyfit function in MATLAB (The Mathworks Inc., Natick, Massachusetts). When increased growth rate was achieved after a defined period of time at a particular concentration, the ionic liquid concentration was increased. This process was repeated until a significant increase in tolerance was achieved. Periodically, samples were frozen in a 25% v/v glycerol solution and stored at − 80 °C for further use.

**Table 1:** Growth phenotypes for *E. coli* K-12 MG1655 and DH1 evolved populations endpoints on ILs ([C_4_C_1_Im]Cl and [C_2_C_1_Im][OAc])

Ionic liquid	Strains	Replicate #	Starting conc. %	Initial growth rate (h^−1^)^a^	Average end conc. %	Final growth rate for end conc. (h^−1^)^a^	Change in IL conc. (%)	Total number of flasks
[C_4_C_1_Im]Cl	MG1655	ALE #1	1.5	0.3	6.2	0.2 ± 0.02	4.7	62
	MG1655	ALE #2	1.5	0.2	6.2	0.1 ± 0.03	4.7	67
	MG1655	ALE #3	1.5	0.3	4.9	0.3 ± 0.12	3.4	63
	MG1655	ALE #4	1.5	0.2	5.6	0.1 ± 0.05	4.1	84
					5.7 ± 0.6	0.2		
	DH1	ALE #5	1.5	0.2	4.8	0.2 ± 0.05	3.3	61
	DH1	ALE #6	1.5	0.2	5.6	0.3 ± 0.08	4.1	72
	DH1	ALE #7	1.5	0.2	5.6	0.1 ± 0.01	4.1	79
	DH1	ALE #8	1.5	0.2	4.2	0.2 ± 0.02	2.7	50
					5.0 ± 0.6	0.2		
[C_2_C_1_Im][OAc]	MG1655	ALE #9	2	0.2	5.9	0.1 ± 0.00	3.9	86
	MG1655	ALE #10	2	0.2	5.9	0.1 ± 0.04	3.9	87
	MG1655	ALE #11	2	0.2	6.5	0.2 ± 0.00	4.5	91
	MG1655	ALE #12	2	0.2	5.9	0.1 ± 0.01	3.9	92
					6.1 ± 0.3	0.1		
	DH1	ALE #13	1	0.1	4.5	0.1 ± 0.06	3.5	77
	DH1	ALE #14	1	0.1	4.5	0.1 ± 0.02	3.5	92
	DH1	ALE #15	1	0.2	4.5	0.2 ± 0.01	3.5	79
	DH1	ALE #16	1	0.2	5.2	0.2 ± 0.02	4.2	88
					4.6 ± 0.3	0.2		

^a^Initial and final growth rates were calculated for the whole population from the first and last 3 flasks of each corresponding population endpoints, respectively

### Primary screening

Evolved isolates were screened for growth properties (growth rate, lag time, and final OD_600_) on selected concentrations (Table 1) of [C_4_C_1_Im]Cl and [C_2_C_1_Im][OAc] using a Growth Profiler (EnzyScreen BV, Leiden, Netherlands). Populations from endpoint evolutions were plated on LB agar plates and ten individual colonies derived from each population were screened at the maximum concentration for which robust growth rates were achieved during the evolution. Colonies from wild type starting strains, *E. coli* K-12 MG1655 and DH1, were used as a control for the primary screening. Selected isolates were inoculated into 500 µL M9 glucose medium in deep well plates and incubated in a plate shaker at 37 °C and 300 rpm shaking. Later, cells were diluted 10× in M9 glucose medium, from which 30 µL was transferred to clear-bottom 96 half-deepwell plates (EnzyScreen BV, Leiden, Netherlands) containing M9 glucose medium with either of the two ionic liquids, such that the final concentration was equal to the concentrations listed in Table 1. Cryogenic stocks of the pre-culture plates were stored in 96-well plates. The half-deepwell plates were incubated at 37 °C with 225 rpm shaking in the Growth Profiler, with scans recorded at 15 min intervals. Green pixel (G) values extracted from the 1 mm diameter circular areas in the center of each well in the images were converted to OD_600_ values using a calibration between OD_600_ (1 cm path-length) and G values. The resulting growth curves for each isolate, Additional file 2: Figure S1, were inspected for those exhibiting robust growth or unique growth profiles such as exhibiting reduced lag-times, increased final densities, and increased in the apparent growth rates. Ten isolates from each population were grouped according to their similarities between these parameters.

### Secondary screening of TALE isolates

Three individual isolates chosen from primary screening from each population underwent secondary screening, where biological replicates were analyzed. The IL concentration was lowered to the average concentration used in the primary screen for all clones. These adjusted IL concentrations for both *E. coli* K-12 MG1655 and DH1 are listed in Additional file 2: Tables S2 and S3, respectively. Selected isolates from the primary screening were steaked out on LB agar from the cryogenic stock plates stored for primary screening. Three individual colonies from each isolate were inoculated as biological replicates into 96-well deepwell plates containing 500 µL M9 glucose and grown overnight. The next day, cryogenic stocks were prepared as described for primary screening. Each well from the overnight culture were inoculated to a low OD_600_ (1:100 dilution) in M9 glucose medium with the specified IL concentration, and growth was monitored until stationary phase was reached. Growth rates were calculated as described previously, and the average values of the three cultures were determined.

### Re-sequencing of improved IL tolerance clones

Sequencing was performed on an Illumina NextSeq (Model 550) Sequencer (San Diego, CA). A total of 45 isolates were re-sequenced. Selected colonies were isolated on LB agar plates, genomic DNA was extracted using PureLink^®^ Genomic DNA Kits (Invitrogen, CA). The quality of extracted DNA was assessed with UV absorbance ratios using a nano drop. Concentration of DNA was quantified using Qubit ds-DNA high sensitivity assay. Paired-end resequencing libraries were generated using a 300 cycle (150 bp × 2) kit from Illumina (San Diego, CA) with loading concentration on Nextseq 1.2 pico-Molar with 1% PhiX spike (Illumina, San Diego, CA) of input DNA total. Re-sequencing data were analyzed using a customized script based on the Breseq version 0.30.1 [14] to map sequence reads and identify mutations relative to the reference strain. The average coverage for each isolate was typically over 400 (a relatively high coverage for clonal sequencing). The genomes of the evolved strains were sequenced and mapped to the genome of the parent strains (NCBI Accession Numbers NC_000913.3 and NC_017625.1) to examine mutations.

### Criteria for choosing the best-performing clones from the secondary screen

Additional file 2: Figure S2 summarizes the method used to choose representative clones from each genotypic-clustered set. These representative clones were being used to screen enhanced performance for each clone. For each genotypic-clustered set, if the physiology was the same with a %RSD (relative standard deviation) ≤ 20% variability in growth rate and final OD, the selection was made based on the clone with the fastest growth rate and highest final OD with least variability. Alternatively, clones with the fastest growth rate and highest final OD were selected.

### Comparing TALE best-performing clones to rational-designed strains

The medium used was a modified M9 glucose containing 4 g/L glucose. The appropriate amounts of antibiotics (100 mg/L carbenicillin and 50 mg/L kanamycin) were added when needed. [C_2_C_1_Im][OAc] (BASF, Germany) was added to the medium as indicated. Seed cultures in LB medium were grown overnight and diluted 1:50 into M9 glucose medium (modified to contain 4 g/L glucose, with 100 mg/L carbenicillin and 50 mg/L kanamycin added when needed) containing varying concentrations of [C_2_C_1_Im][OAc] or [C_2_C_1_Im]Cl (BASF, Germany). Cultures were grown in 96 well plates (BD Falcon) on a Tecan F-200 Pro microtiter plate reader (Maennedorf, Switzerland) or in 250 mL flat-bottom flasks at 200 rpm and 37 °C. The absorbance of samples from 250 mL flat-bottom flasks was measured at 600 nm using a Spectramax M2 spectrophotometer (Molecular Devices, USA). Initially, selected clones were screened in [C_2_C_1_Im][OAc] to determine levels of tolerance to a IL not used for the ALE process (i.e., cross-tolerance), then best-performing clones were used for the comparison in [C_2_C_1_Im][OAc] and [C_2_C_1_Im]Cl in either M9 or LB medium to understand performance in both a minimal defined and rich undefined media. The parent strain *E. coli* K-12 MG1655 was used as a control. The IL tolerant strains JBEI-10101 [8] and JBEI-13314 [12] were also tested for comparison.

## Results

A tolerance adaptive laboratory evolution (TALE) experiment was utilized to generate strains which could tolerate toxic concentrations of similarly close ionic liquids (ILs) and identify mutations which likely confer a fitness advantage under more economically advantageous bioprocessing conditions, i.e., pretreated biomass solution containing some ILs. Two different *E. coli* strains were chosen for the study (K-12 MG1655 and DH1) as well as two types of ILs; 1-butyl-3-methylimidazolium chloride ([C_4_C_1_Im]Cl) and 1-ethyl-3-methylimidazolium acetate ([C_2_C_1_Im][OAc]). The *E. coli* strains were chosen as they are often used in bioprocessing applications [15, 16] and as the adaptive responses of K-12 MG1655 toward minimal medium growth is known [17]. IL pretreatment for biomass deconstruction has been demonstrated in several studies as a promising approach to solubilize cellulosic polysaccharides, thereby increasing the enzymatic turnover of saccharification, and also reducing the formation of inhibitory by-products [6, 18, 19].

### Description of fitness changes during the TALE experiment

**Fig. 1:**
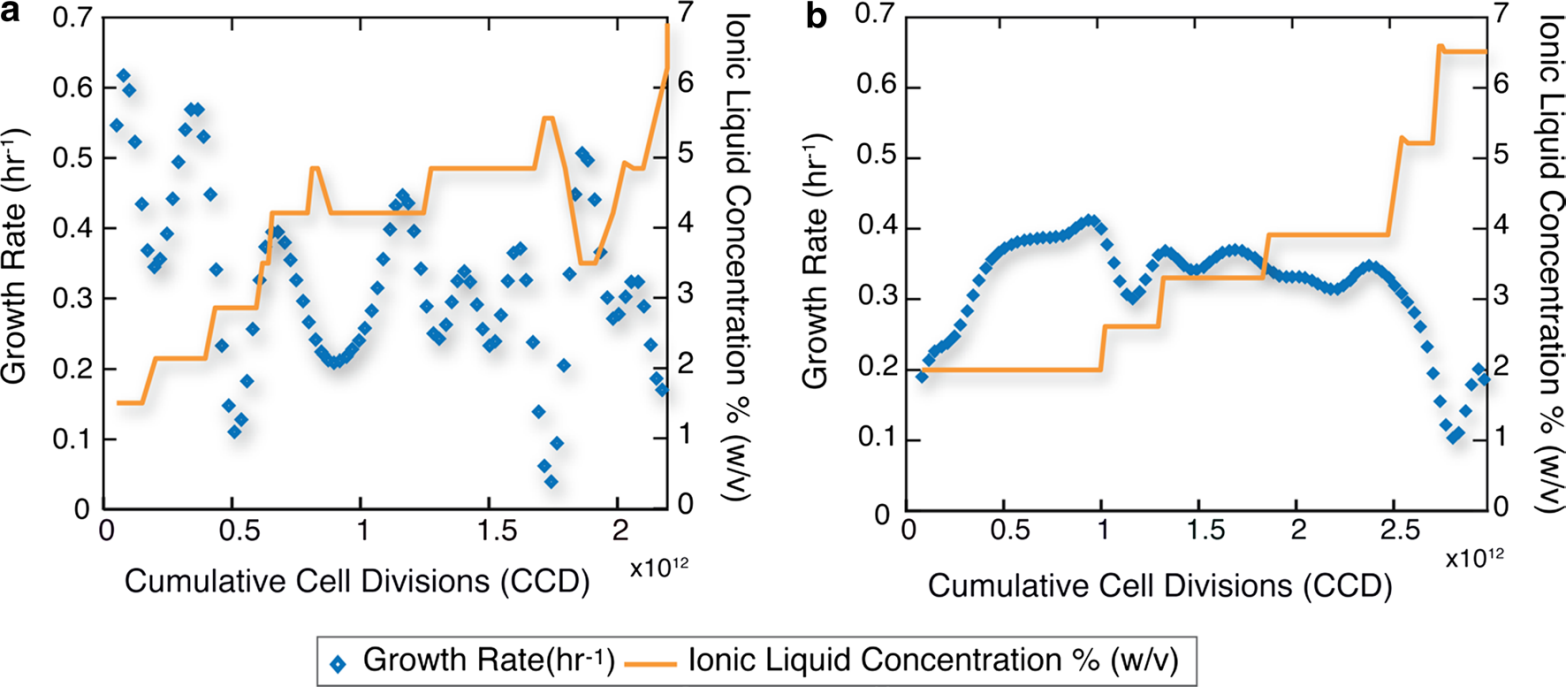
Plots of population growth rate versus IL concentration over the course of two TALE experiments for increasing IL tolerance. **a** *E. coli* K-12 MG1655 population ALE #1 evolved on glucose minimal medium with ([C_4_C_1_Im]Cl) and, **b** *E. coli* DH1 population ALE #11 evolved on glucose minimal medium with ([C_2_C_1_Im][OAc]). Depicted are fitness trajectories and IL concentration versus cumulative cell divisions (CCD) experienced by the cultures for two out of the total sixteen individual experiments. IL concentration was increased step-wise when the growth rate for the population increased

The process of TALE was successful in generating strains with increased tolerance to ILs. Four replicate populations of each *E. coli* strain were evolved on each of the ILs. The IL concentration was increased under continuous exponential batch growth over the course of the experiment when an observed fitness over a threshold was achieved (i.e., a growth rate of ≥ 0.15/h). During this period of time, each of the populations underwent a total number of cumulative cells divisions (CCD) of 1.93 × 10^12^, 2.73 × 10^12^ for *E. coli* K-12 MG1655 and 1.56 × 10^12^, 1.61 × 10^12^ CCD for DH1 populations with [C_4_C_1_Im]Cl and [C_2_C_1_Im][OAc], respectively (Additional file 2: Table S4). The use of CCD has previously been shown to be a meaningful measure of evolution time [20]. The observed growth rates during a representative TALE experiment as well as the initial and final IL concentrations are shown in Fig. 1. Similar plots for the remaining populations are shown in Additional file 2: Figure S3.

The ability of K-12 MG1655 populations to adapt to increasing IL concentrations was superior to DH1 for both ionic liquids, with average final concentrations achieved of 5.7 ± 0.6% (w/v) and 6.1 ± 0.3% (w/v) for MG1655 and 5.0 ± 0.6% (w/v) and 4.6 ± 0.3% (w/v) for DH1, with [C_4_C_1_Im]Cl and [C_2_C_1_Im][OAc], respectively (Table 1). During the TALE experiments, population fitnesses fluctuated in response to the concentration of IL added to media (Fig. 1). Additionally, applied IL concentration increases were sometimes too large and resulted in ceased growth. In these instances, the concentration was adjusted back to the previous concentration in order to restore growth, and a smaller step change in concentration was employed. Overall, there were approximately five increases in IL concentration for each experiment using an average step increase of 0.75% (w/v) over the current concentration. Each experiment contained an average of 67 flasks in [C_4_C_1_Im]Cl and an average of 87 flasks in [C_2_C_1_Im][OAc]. Screening of the evolved populations was subsequently performed to understand the overall tolerance and performance of evolved isolates.

### Screening of evolved isolates for improved tolerance

Isolates from evolved populations were screened for improved tolerance to ILs and to help identify causal mutations through genotype–phenotype relationships following resequencing. A primary screen was performed to establish whether selected isolates from each population (10 isolates from each of 16 populations) could grow reproducibly in the average final IL concentration achieved during TALE (Table 1). From this analysis, three isolates from each population were selected for secondary screening and whole genome sequencing based on qualitative differences in the observed primary screen growth rates (Additional file 2: Figure S1). Additionally, isolates were selected that still exhibited tolerance but displayed unique growth phenotypes. Clones from one of the populations of DH1 on [C_4_C_1_Im]Cl did not grow; therefore, this population was dropped from the analysis (ALE #8). A total of 45 isolates were whole genome resequenced.

### Whole genome resequencing and mutation analysis

Whole genome resequencing was used to determine the genetic basis of fitness tolerance phenotypes. Key mutations were determined by comparing all of the clones and identifying genes, or genetic regions (i.e., intergenic regions) which had multiple unique mutations or were mutated across isolates from independent populations. Overall, there were 37 and 53 unique mutations identified for *E. coli* K-12 MG1655 and DH1, respectively. Each isolate had between 1 and 13, or 2 and 12 mutations identified for MG1655 or DH1, respectively. There were three hyper-mutator isolates (1 from MG1655 and 2 from DH1) identified. The MG1655 isolate had 267 mutations, while the DH1 isolates had 39 ± 4 mutations, as compared to the average mutations 5 ± 4 for the non-hypermutating clones from both strains with standard deviation shown between replicates. The hyper-mutator clone from MG1655 had two mutations in two different SOS genes, *uvrA* and *uvrC*, which are involved in DNA repair processes under stressed conditions [21] as well as an intragenic IS element mutation between *fnr* and *ogt*, where the later gene is a methyltransferase known to be involved in hypermutating phenotypes [22]. For the two DH1 hypermutator clones, it was not apparent which genes may have caused such phenotypes. Hypermutating clones were excluded from further analysis to simplify the genetic analysis and as they have a greater potential for instability when utilizing them as a platform strain.

Key mutations are presented in Table 2, which are defined as mutations in genes or regions that were found to be repeatedly mutated across different isolates of K-12 MG1655, DH1, or in both strains. A full summary of mutations for each isolate are given in Additional file 1. The mutations were categorized as ‘combined’ or ‘strain-specific’. Overall, there were two genetic regions identified as ‘combined’ mutations occurring in both strains. Further, there were four and six strain-specific key mutations for MG1655 and DH1, respectively.

**Table 2:** Key mutations categorized by those which repeatedly mutated (i.e., had multiple unique mutations in any ORF or genetic region) in K-12 MG1655, DH1, or those which were shared across the strains

Strain	Gene	Mutation	Mutation type	Mutated allele function	Strain	IL observed	Count
Combined	mdtJ/tqsA	Intergenic (− 56/− 237) Δ120 bp	DEL	Transporter	MG	B, E	23
	tqsA/mdtJ	Intergenic (− 239/− 54) Δ120 bp	DEL	Transporter	DH1	B	5
	tqsA	Coding (857–868/1035 nt) Δ12 bp	DEL	Transporter	MG	B	6
	pntA–tqsA	Δ3035 bp	DEL	Transhydrogenase/transporter	DH1	B	2
	yhdP	Coding (2440–2443/3801 nt) Δ2 bp	DEL	Transporter	MG	B	1
		Coding (647/3801 nt) (TGGAGCC)1 → 2	INS	Transporter	MG	B	4
		Coding (200–201/3801 nt) IS5 (−) +4 bp	MOB	Transporter	MG	B	1
		Coding (3102–3110/3801 nt) IS element(+) +9 bp	MOB	Transporter	DH1	B	1
		Coding (2887–2890/3801 nt) Δ4 bp	DEL	Transporter	DH1	B	1
MG1655	rpoC	P359L (CCA → CTA)	SNP	RNA synthesis	MG	B	4
		F773Y (TTC → TAC)	SNP	RNA synthesis	MG	B	6
		R1075S (CGT → AGT)	SNP	RNA synthesis	MG	B	1
	cspC	Coding (33–41/210 nt) IS1 (−) +9 bp	MOB	Stress protein	MG	B	1
		Q37* (CAG → TAG)	SNP	Stress protein	MG	E	1
	rpsG	Coding (460/540 nt) Δ1 bp	DEL	Subunit of ribosome	MG	B	1
		L157* (TTA → TGA)	SNP	Subunit of ribosome	MG	B	2
	rph	Pseudogene (667/669 nt) + C	INS	Nucleotide biosynthesis	MG	E	2
	pyrE/rph	Δ82 bp	DEL	Nucleotide biosynthesis	MG	B	5
DH1	rho	G61E (GGA → GAA)	SNP	Transcription termination factor	DH1	B	2
		Y80H (TAC → CAC)	SNP	Transcription termination factor	DH1	E	5
		Y80C (TAC → TGC)	SNP	Transcription termination factor	DH1	B	2
		T406P (ACC → CCC)	SNP	Transcription termination factor	DH1	E	4
	fhuA	Coding (337–479/2244 nt) Δ143 bp	DEL	Transport of ferrichrome	DH1	E	1
		Coding (1442/2244 nt) (GTCATAACGACCGCCTAGGG)1 → 2	INS	Transport of ferrichrome	DH1	E	1
		Coding (2107/2244 nt) Δ1 bp	DEL	Transport of ferrichrome	DH1	B	2
		Coding (2129/2244 nt) Δ1 bp	DEL	Transport of ferrichrome	DH1	E	4
	rcdA	L55S (TTG → TCG)	SNP	Transcription regulator	DH1	E	1
		Coding (338–341/537 nt) IS element(+) +4 bp	MOB	Transcription regulator	DH1	E	1
	purB	K404T (AAG → ACG)	SNP	Nucleotide biosynthesis	DH1	B	2
		S21N (AGC → AAC)	SNP	Nucleotide biosynthesis	DH1	E	1
	gadE	Coding (273–281/528 nt) IS element(-) +9 bp	MOB	Transcriptional activator	DH1	E	2
		Coding (273–281/528 nt) IS element(+) +9 bp	MOB	Transcriptional activator	DH1	E	1

Different unique mutations in the same gene or allele were identified in different clones across the different experiments. The mutations were categorized as ‘combined’, i.e., identified in both strains, or ‘strain-specific’. B and E denotes [C_4_C_1_Im]Cl and [C_2_C_1_Im][OAc] IL, respectively, where MG denotes K-12 MG1655 strain

The first key mutation occurring in both strains was in the non-coding intergenic region between *mdtJ* and *tqsA*. Mutations in this region have been previously reported to improve tolerance of *E. coli* toward isobutanol [23]. The *mdtJ* gene encodes a component of a multidrug efflux pump that also physiologically exports spermidine [24]. Surprisingly, an identical deletion of Δ120 bp in the intergenic region, i.e., *mdtJI* promoter region, between the genes occurred in both of the strains. This deletion was found in every MG1655 clone isolated. The widespread penetration of this mutation could be due the fact that this deletion was the easiest to loop out under IL or other stress conditions [23] or could be due to occurrence of this mutation in the seeding culture for the experiment (although it did occur in both K-12 MG1655 and DH1). The other types of mutations in this region were structural changes in the *tqsA* gene—one was an intragenic in-frame ∆12 bp deletion, and the other was a ∆3035 bp deletion which included the *pntB*, and *pntA* genes located next to *tqsA* on the chromosome. The latter of these mutations is likely a loss of function mutation for *tqsA*. *PntA* and *pntB*, encode for the two subunits forming pyridine nucleotide transhydrogenase enzyme [25] and are important for redox balance in the cell [26, 27]. The *tqsA* gene encodes a transporter of quorum-sensing signal AI-2 which plays a role in control of biofilm formation in *E. coli* K-12 by enhancing transport of autoinducer-2 (AI-2) [28]. A Δ*tqsA* mutant was found to carry higher resistance to various drugs [28], which reveals a potentially tolerance role in the evolved strains in this study.

The second key mutation occurring in both strains was in *yhdP*, a gene encoding a putative transport protein [29]. A total of five unique mutations, all structural changes, were identified in *yhdP*—three in MG1655 and two in DH1. These mutations were two out-of-frame short deletions, two IS mobile element insertions, and a short 7 bp duplication. All of these structural mutations suggest a loss of function. There are no previous studies examining the role of *yhdP* in tolerance, to the best of our knowledge, making this finding a novel discovery.

Strain-specific key mutations (Table 2) were also identified in MG1655 and DH1. In MG1655, three different coding mutations were identified in *rpoC*, encoding the β′ subunit of RNA polymerase. Prior ALE studies have identified *rpoC* coding mutations, which were found to both boost metabolic efficiency in glucose minimal medium [30] and improve growth at 42 °C [31, 32]. Probable loss-of-function mutations (premature stop codon and IS element insertion) were identified in *cspC* (encoding a stress protein of the CspA family). CspC is thought to stabilize *rpoS* mRNA when overexpressed [33] and to have activity as a transcription anti-terminator [34]. Mutations in this gene were previously found to play a role in stress responses [33]. Two different mutations occurred in the *rpsG* gene, encoding the essential S7 subunit of the 30S ribosome. These were a Δ1 bp deletion and a premature stop codon near the end of the gene. These mutations likely correct a defective 23 amino acid C-terminal extension to RpsG that occurs only in K-12 derived strains and that causes increased degradation of this protein. Similar mutations have previously been observed in MG1655 evolved for increased tolerance toward sodium cation [35]. Truncation of *rpsG* is thus likely a general stress coping mechanism. An ∆82 bp deletion was also found in the intragenic region between *pyrE* and *rph* and an insertion in *rph* was observed seven times in different clones. The *rph* gene encodes for an RNase PH [36], where *pyrE* encodes an orotate phosphoribosyltransferase [37]. Related deletion mutations were reported in different ALE studies including adaptation to lactate, minimal glucose medium, and high temperature (42 °C) [17, 30, 32]. The wild type strain *E. coli* K-12 has a frameshift mutation in *rph* which leads to pyrimidine starvation on minimal media due to resulting low levels of orotate phosphoribosyltransferase encoded by *pyrE* [38]. It appears that these mutations can be attributed to a 15% growth advantage by alleviation of defects in pyrimidines biosynthesis [39], and these mutations are predominantly general adaptations to growth on minimal medium. Interestingly, in DH1, mutations were not found in *rpoC*, *rpsG*, or *pyrE*/*rph*, with mutations in the latter two regions serving to correct metabolic and ribosomal protein defects that are present in all K-12 strains, including DH1.

Strain-specific mutations in DH1 included genes involved in the processes of transcriptional activation and transportation. In 13 isolates, *rho*, encoding the Rho transcription terminator with annotated function as transcription termination factor *Rho* [40], all contained coding SNPs. Coding mutations in Rho have previously been observed as a major contributor to ethanol tolerance [41, 42], and have been found to reduce the rate of Rho-dependent transcription termination in an ethanol-tolerant mutant [42]. The *fhuA* gene (encoding a ferrichrome outer membrane transporter) had several unique mutations in eight different isolates, including two unique ∆1 bp deletions, a ∆143 bp deletion, and a 20 bp short insertion. Additionally, two unique mobile element insertions were found between three isolates in *gadE* (encoding the GadE transcriptional activator), and a coding SNP and a mobile element insertion were found in two isolates in *rcdA* (encoding the RcdA transcriptional activator). Interestingly, deletion of *rcdA* was previously found to improve tolerance of DH1 toward IL [12]. Finally, two different coding SNPs in *purB* (encoding adenylosuccinate lyase) were found in three isolates.

### Secondary screening of the evolved clones

A secondary screen was performed to generate quantitative data on the resequenced isolates after their genetic bases had been determined (see “Methods”). Resequenced isolates were clustered (see “Methods”) based on their genotypes in order to assess their performance into three groups: genetically-identical where clones share identical genotypes; genetically-similar based on shared mutations (an expected outcome as multiple clones were isolated from the same population); and hyper-mutator isolates—which were eliminated from the secondary screening and further analysis. Overall, there were 3, 3, 2, and 3 genetically-similar clusters for the MG1655/[C_4_C_1_Im]Cl, DH1/[C_4_C_1_Im]Cl, MG1655/[C_2_C_1_Im][OAc], and DH1/[C_2_C_1_Im][OAc] conditions, respectively. Most of the clones showed reproducible performance when culturing. Results for both *E. coli* K-12 MG1655 and DH1 are summarized in Additional file 2: Tables S2 and S3, respectively. A few isolates did not grow during the secondary screen for unknown reasons (Additional file 2: Table S5).

Isolated strains from the study with similar genotypes exhibited a similar performance when tolerating ILs. A main difference in this study was that the growth rate criterion was used to quantify strains with improved performance. The coefficients of variation in growth rate (h^−1^) between isolates that were genetically-identical or genetically-similar were 21 and 11%, respectively. Some of the resequenced isolates exhibited no growth (6 out of 45 clones, 4 with similar genotypes, Additional file 1). This non-growth could be a result of moving from unstressed to a highly-stressed condition during the screen, but it was not explored further. A more detailed analysis of the secondary screening results is provided in Additional file 2. The most promising isolates (based on criteria in Additional file 2: Figure S2) from each genetically identical or similar cluster were selected for further testing and are provided in Table 3.

**Table 3:** Selected clones from each genetically identical or similar cluster were selected for testing for tolerance to [C_2_C_1_Im][OAc] and [C_4_C_1_Im]Cl ionic liquids

TALEs	IL type	Concentration (%)	Gene set	Aver. growth rate (h^−1^)	Aver. final OD_600_	Aver. lag-time (h)
MG 4.7	[C_4_C_1_Im]Cl	5.4	MG-BM-3A	0.26 ± 0.024	0.87 ± 0.039	4.88 ± 0.04
MG 3.10	[C_4_C_1_Im]Cl	5.4	MG-BM-3D	0.31 ± 0.009	0.94 ± 0.04	10.85 ± 1.24
MG 4.5	[C_4_C_1_Im]Cl	5.4	MG-BM-3E	0.30 ± 0.015	0.84 ± 0.082	3.19 ± 1.17
MG 11.10	[C_2_C_1_Im][OAc]	4.6	MG-EM-1	0.23 ± 0.003	1.69 ± 0.004	1.55 ± 0.006
MG 12.7	[C_2_C_1_Im][OAc]	4.6	MG-EM-1	0.27 ± 0.005	1.1 ± 0.03	1.9 ± 0.015
MG 10.9	[C_2_C_1_Im][OAc]	4.6	MG-EM-1A	0.09 ± 0.003	0.74 ± 0.004	1.99 ± 0.006
DH1 5.3	[C_4_C_1_Im]Cl	4.6	DH-BM-3A	0.32 ± 0.005	0.96 ± 0.01	12.51 ± 0.075
DH1 5.10	[C_4_C_1_Im]Cl	4.6	DH-BM-2	0.58 ± 0.023	0.61 ± 0.12	8.8 ± 1.059
DH1 6.7	[C_4_C_1_Im]Cl	4.6	DH-BM-1	0.36 ± 0.033	0.99 ± 0.094	5.62 ± 0.154
DH1 7.5	[C_4_C_1_Im]Cl	4.6	DH-BM-1	0.25 ± 0.025	1.39 ± 0.014	11.49 ± 3.604
DH1 13.10	[C_2_C_1_Im][OAc]	4.2	DH-EM-2A	0.14 ± 0.013	0.52 ± 0.385	9.59 ± 0.66
DH1 14.2	[C_2_C_1_Im][OAc]	4.2	DH-EM-2B	0.2 ± 0.027	0.36 ± 0.304	4.91 ± 1.083
DH1 13.8	[C_2_C_1_Im][OAc]	4.2	DH-EM-1	0.23 ± 0.007	0.6 ± 0.085	8.57 ± 3.393
DH1 15.2	[C_2_C_1_Im][OAc]	4.2	DH-EM-3C	0.28 ± 0.057	0.57 ± 0.148	6.33 ± 0.259
DH1 16.7	[C_2_C_1_Im][OAc]	4.2	DH-EM-3A	0.31 ± 0.014	0.26 ± 0.172	5.34 ± 0.44

Each of the TALE-derived isolates is presented with the corresponding IL-type and concentration in which it was originally evolved along with phenotypic characteristics of each of the selected isolates

### Tolerance testing of the selected evolved strains and comparison to previous work

**Fig. 2:**
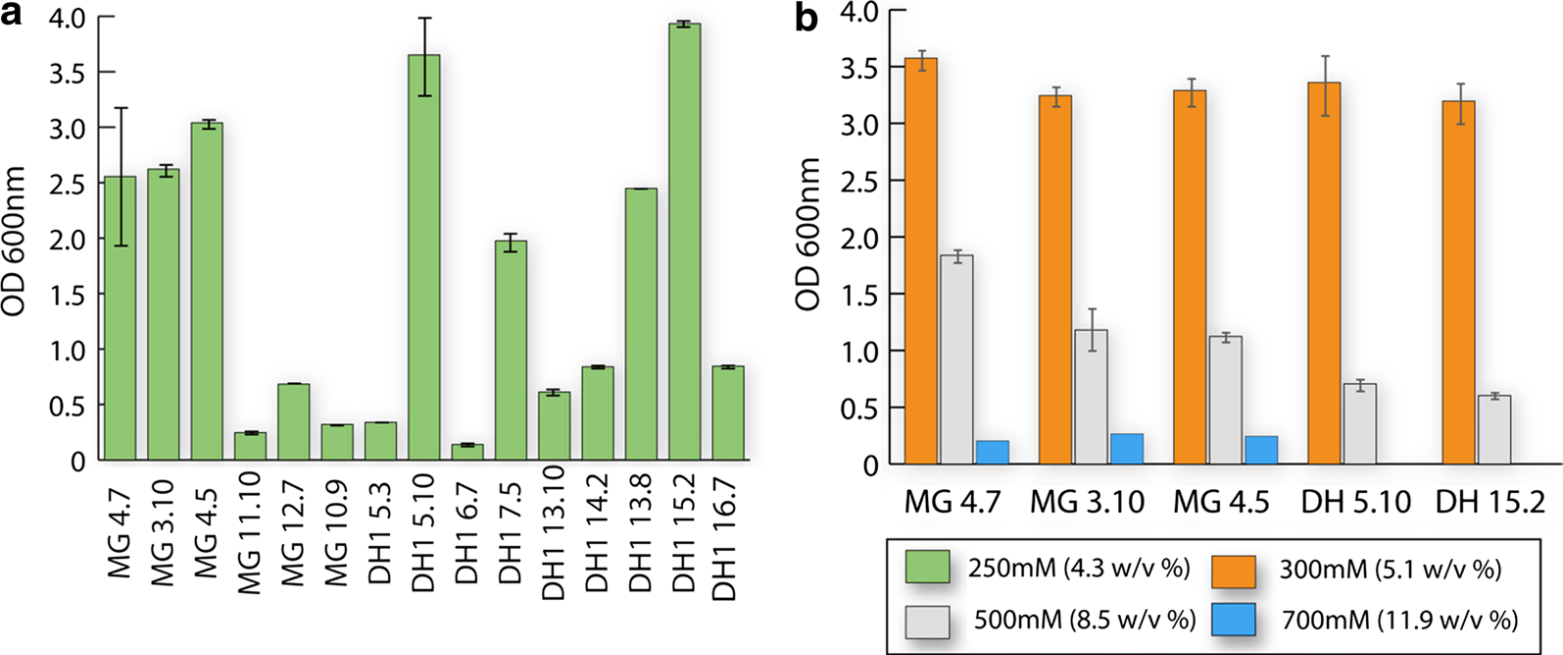
Performance of evolved clones in batch culture under M9 minimal media conditions with various [C_2_C_1_Im][OAc] IL loadings. Shown is the final optical density (OD_600nm_) of the selected best performing clones (Table 3) under different concentrations of [C_2_C_1_Im][OAc]: **a** first, a screen with 250 mM and all best performing clones, **b** second, a follow up screen with 300, 500 and 700 mM utilizing the highest final density clones from the 250 mM screen. Surprisingly, a number of clones that were originally evolved for [C_4_C_1_Im]Cl tolerance showed high cross-tolerance to elevated [C_2_C_1_Im][OAc] concentrations. At the highest concentration (700 mM), only the MG1655 derived strains showed measurable growth

Fifteen isolates selected from the secondary screen results (Table 3) were tested for tolerance to [C_2_C_1_Im][OAc], an ionic liquid with arguably the best characteristics for lignocellulose solubilization and pretreatment [5]. This screen was performed to establish quantitative differences in final cellular densities achieved in batch culture (Fig. 2) and to understand cross-tolerance to other imidazolium-based ILs.

While almost all the isolates were capable of growth in M9 minimal medium in the presence of 4.3% (w/v) (250 mM) [C_2_C_1_Im][OAc] (Fig. 2a), a few isolates had significantly higher final densities in this condition. These high performing isolates were three K-12 MG1655 (MG 4.7, MG 3.10, and MG 4.5) and two DH1 (DH 5.10 and DH 15.2) derivatives. Interestingly, only one of the five best performers were actually evolved on [C_2_C_1_Im][OAc], DH1 15.2, while the remainder were isolated from the [C_4_C_1_Im]Cl evolutions. Furthermore, increased amounts of [C_2_C_1_Im][OAc] were tested with the five best performing isolates (Fig. 2b). Increasing amounts of IL inhibited growth in all the strains, but robust growth (i.e., a final OD_600_ > 0.5) was detected for all five clones at 8.5% (w/v) IL and detectable growth was observed for the MG clones, MG 4.7, MG 3.10, and MG 4.5, in concentrations up to approximately 11.9% (w/v) (700 mM) [C_2_C_1_Im][OAc] (Fig. 2b). These tests demonstrated that the evolved isolates display cross tolerance to ILs which they were not exposed to during the ALE process and the levels of IL tolerated were impressive when compare to previously-developed strains.

The final concentration of ILs tolerated by evolved isolates using TALE were compared to previously reported strains generated for IL tolerance (Table 4). The robust growth of the best performing clones MG 4.7, MG 3.10, DH 5.10 and DH 15.2 observed at 8.5% (w/v) compares favorably with other reported values for engineered and evolved strains. For example, the tolerance achieved from [12] was based on introducing a mutation in the transcriptional regulator encoded by *rcdA* with tolerance up to 3% (w/v) [C_2_C_1_Im][OAc] achieved in LB medium. Additionally, thermophilic communities have been isolated by enriching them for tolerance to [C_2_C_1_Im][OAc], which has resulted in the identification of a mixed population tolerant to 6% w/v [C_2_C_1_Im][OAc] [10]. Finally, an ALE approach had also been previously employed to develop a strain tolerant to [C_4_C_1_Im]Cl of approximately 7% (w/v) in rich media (LB) [11]. A direct experimental comparison to two previously developed strains was also conducted.

**Table 4:** Comparison of IL tolerance in the generated TALE evolved strains in the current study and previously reported tolerances from different studies with each respective tolerant biological system

Strain	IL’s type	Description/mechanism	Culturing environment	Concentration % (w/v) criteria	References
*E. coli K-12 MG1655* ∆*fadD* mutant	[C_4_C_1_Im]Cl	ALE (90 days long)	LB broth	7.0	[11]
*E. coli* DH1	[C_2_C_1_Im]Cl	Efflux pump encoded by *eilA* from *Enterobacter lignolyticus*	Minimal media (M9)	5.8	[9]
JBEI-10101 (*E. coli DH1*)	[C_2_C_1_Im]Cl	Native *eilA* pump from *Enterobacter lignolyticus*	Minimal media (M9)	5.5	[8]
Thermophilic communities	[C_2_C_1_Im][OAc]	*Thermophilic* enrichment	Raw material with minimal media	6.0	[10]
JBEI-13314 (*E. coli* DH1 rcdA mutant)	[C_2_C_1_Im][OAc]	Mutation of transcriptional regulator encoded by rcdA	LB broth	3.0	[12]
*E. coli* K-12 MG1655 mutants MG4.7, MG 3.10, and MG 4.5	[C_2_C_1_Im][OAc]	TALE isolate	Minimal media (M9)	8.5	This work
*E. coli* DH1 mutants DH 5.10 and 15.2	[C_2_C_1_Im][OAc]	TALE isolate	Minimal media (M9)	8.5	This work

The high performing isolates from this work were three K-12 MG1655 mutants (MG 4.7, MG 3.10, and MG 4.5) and two DH1 mutants (DH 5.10 and DH 15.2)

### Comparison of selected evolved strains to previously-developed tolerant strains

The best performing IL tolerant isolates, MG 4.7 and MG 3.10, were compared to rationally-engineered IL tolerant strains, JBEI-10101 [8] and JBEI-13314 [12], to provide a direct comparison for the efficacy of the evolution process as compared to rational engineering approaches. JBEI-10101 [8] is DH1 harboring a plasmid containing genes for an MFS-1 pump from *Enterobacter lignolyticus* and its response regulator (*eilAR*), and the JBEI-13314 [12] is DH1 carrying a deletion in *rcdA*, which encodes a predicted transcriptional regulator of the MFS-1 pump *ybjJ*. Two different media types were used in this comparison: a rich undefined LB medium and a minimal defined M9-glucose medium. This comparison was performed using two different promising IL compounds, 300 mM of either [C_2_C_1_Im][OAc] [5.1% (w/v)] or [C_2_C_1_Im][Cl] [4.4% (w/v)]. [C_2_C_1_Im][Cl] which contains a chloride anion, was found to be effective towards dissolving cellulose in comparison to larger anions, [C_2_C_1_Im][OAc] [43]. Furthermore, ILs with anions such as acetate (e.g., [C_2_C_1_Im][OAc]) have lower viscosities and this is beneficial as it facilitates the dissolution process [9].

**Fig. 3:**
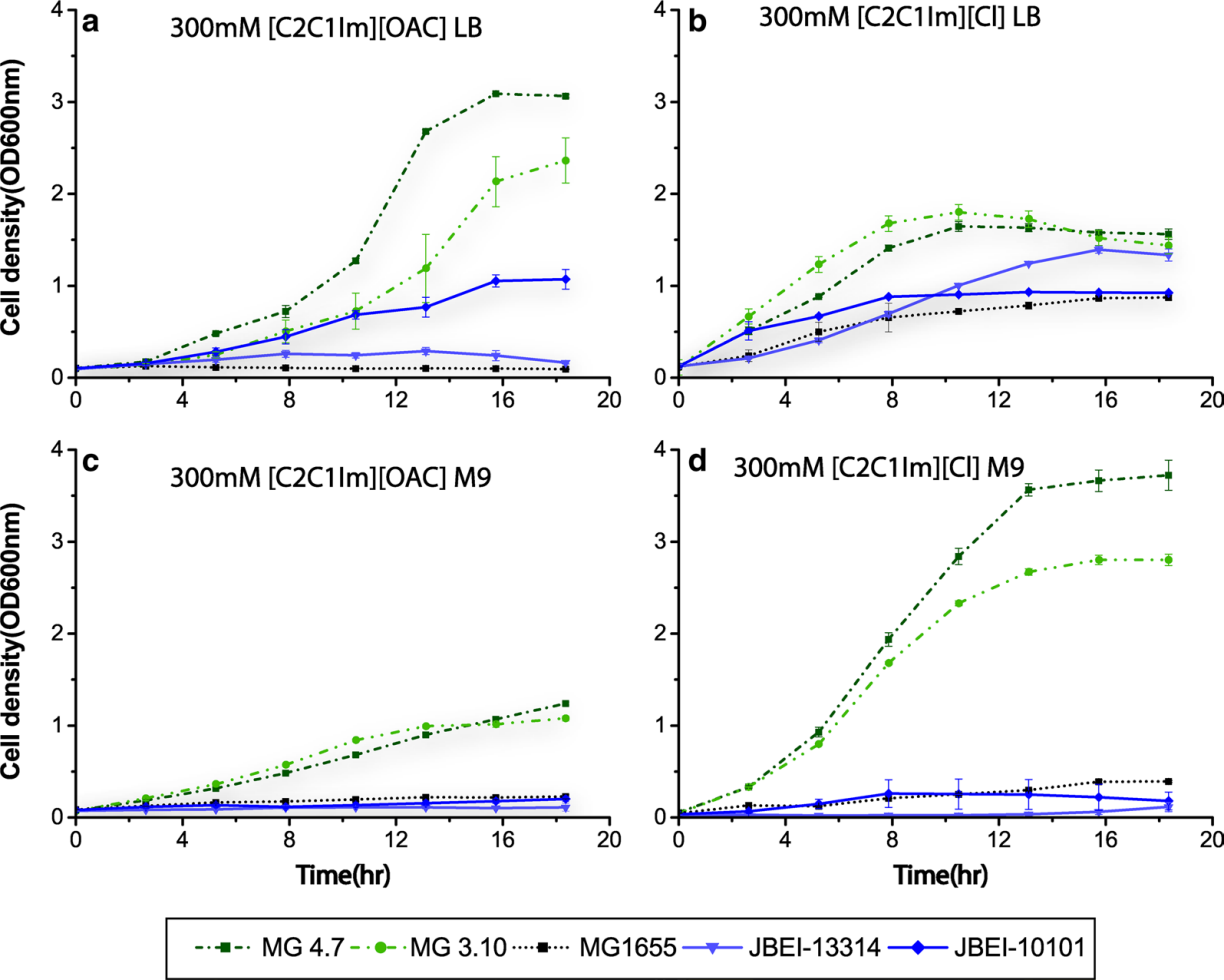
Comparison of TALE evolved IL tolerant clones MG1655#4.7 and MG1655#3.10 to previously engineering tolerant strains JBEI-13314 and JBEI-10101 in LB (**a**, **b**) and M9 (**c**, **d**) media containing either 300 mM of [C_2_C_1_Im][OAc] [5.1% (w/v)] or [C_2_C_1_Im][Cl] [4.4% (w/v)]. TALE evolved clones exhibited improved growth compared to rationally-designed strains, particularly in M9, where JBEI-13314 and JBEI-10101 were severely inhibited

The performance of the TALE derived strains was superior to those developed through rational engineering. In LB medium at 300 mM of [C_2_C_1_Im][OAc] or [C_2_C_1_Im]Cl, the performance of JBEI-13314 and JBEI-10101 was improved over the background control of a wild-type MG1655. In the same conditions, the TALE-derived strains, MG 4.7 and MG 3.10, grew at a significantly faster rate and to a higher final density than the rationally engineered strains. Specifically, the TALE derived strain, MG 4.7, grew to a final OD_600_ of approximately 3.0 and 1.5 in LB containing [C_2_C_1_Im][OAc] and [C_2_C_1_Im]Cl, respectively, versus final ODs of approximately 1.0 on both ILs for the best performing rationally engineered JBEI-10101 strain. Similarly, the growth rates were higher for the TALE-derived strains (Fig. 3a, b). It should be noted that the TALE-derived strains had not been evolved in LB, whereas the JBEI strains were benchmarked in LB (or similar rich medium) as a base medium [8, 12]. Further, in M9 glucose medium containing 300 mM [C_2_C_1_Im][OAc] or 300 mM [C_2_C_1_Im]Cl, the final cell biomass, OD_600_, values reached were approximately, 1.5 and 4.0 for MG 4.7 and 1.0 and 3.0 for MG 3.10 in [C_2_C_1_Im][OAc] and [C_2_C_1_Im]Cl, respectively (Fig. 3c, d), whereas JBEI-13314 and JBEI-10101 exhibited virtually no detectable growth.

The tolerance phenotypes demonstrated by the TALE-derived isolates gives a strong indication of the causality of the mutations identified in this work, specifically, the ‘combined’ key mutations (Table 2). Both the MG 4.7 and MG 3.10 were found to carry ten mutations each, eight being identical and shared, and several mutations were from the combined key mutation set. Namely, MG 4.7 and MG 3.10 share a ∆120 bp deletion in the intergenic region between *mdtJ*/*tqsA* and 12 bp deletion in *tqsA*. Both strains also carry an additional key mutation, a coding SNP in *rpoC*. Further, they both carry different frameshift mutations in the *yhdP* gene. Differentiating key mutations are a ∆1 bp deletion in *rpsG* in MG 3.10 isolate, and a coding SNP in *rpsA* in MG 4.7, with both genes encoding ribosomal protein subunits.

The best performing DH1 isolates, DH1 5.10 and DH1 15.2, also shared key mutations. Each isolate possessed seven mutations overall, none of them identical between the isolates. However, shared genes that were mutated included coding SNPs in the *rho* gene. Other strain-specific key mutations were identified in the strains including SNPs in *purB* and *cspC* identified in DH1 5.10 and a frameshift insertion mutation in *fhuA* in DH1 15.2. It should be noted that the gene encoding the regulator of *purB*, *purR* [44], carried a mutation in the isolate that did not possess a direct mutation in *purB*. Thus, the mutations identified as key in Table 2 can be linked to high performing phenotypes and are likely causal. However, detailed studies to reveal their mechanism of causality are required.

## Discussion

The economical and efficient break down of lignocellulosic material into carbon feedstocks is an essential step in renewable bioprocessing. Ionic liquid (IL) solubilization is a promising method for breakdown of lignocellulosic material, however these compounds are toxic to most bioproduction chassis strains. Thus, the scope of this study was to generate IL tolerant strains utilizing an adaptive laboratory evolution process. Accordingly, the main contributions from this work are: (1) effective generation of IL tolerant strains (including cross-tolerance) for two common production chassis, *E. coli* K-12 MG1655 and DH1, which can be used as platform strains for utilizing feedstocks generated through IL degradation methods, (2) insights into both strain-specific and global mechanisms of IL tolerance through examining key mutations found in multiple parallel evolved isolates, and (3) establishing a viable method using a multiple population TALE approach with next-generation sequencing towards generating tolerant strains. This method was benchmarked via comparison to rational engineering approaches.

TALE was successful in generating strains that were tolerant to the targeted ILs. After approximately 40 days of continuous exposure to ILs during growth (mostly in exponential growth), populations of cells were able to grow at approximately threefold or greater of the initial concentration of each IL compared to the wild type (Table 1). Tolerance levels of isolated clones are impressive when compared to other tolerant bacterial strains [8, 12, 45]. Additionally, the selected best performing strains demonstrated high level of IL cross-tolerance toward [C_2_C_1_Im][OAc]; detectable growth at 11.9% (w/v) for TALE-derived *E. coli* K-12 MG1655 clones and robust growth at 8.5% (w/v) for the same MG1655 isolates plus the *E. coli* DH1 best-performing clones. Thus, the TALE-derived strains show promise as platform strains for utilization of biomass hydrolysates generated using IL treatment.

**Fig. 4:**
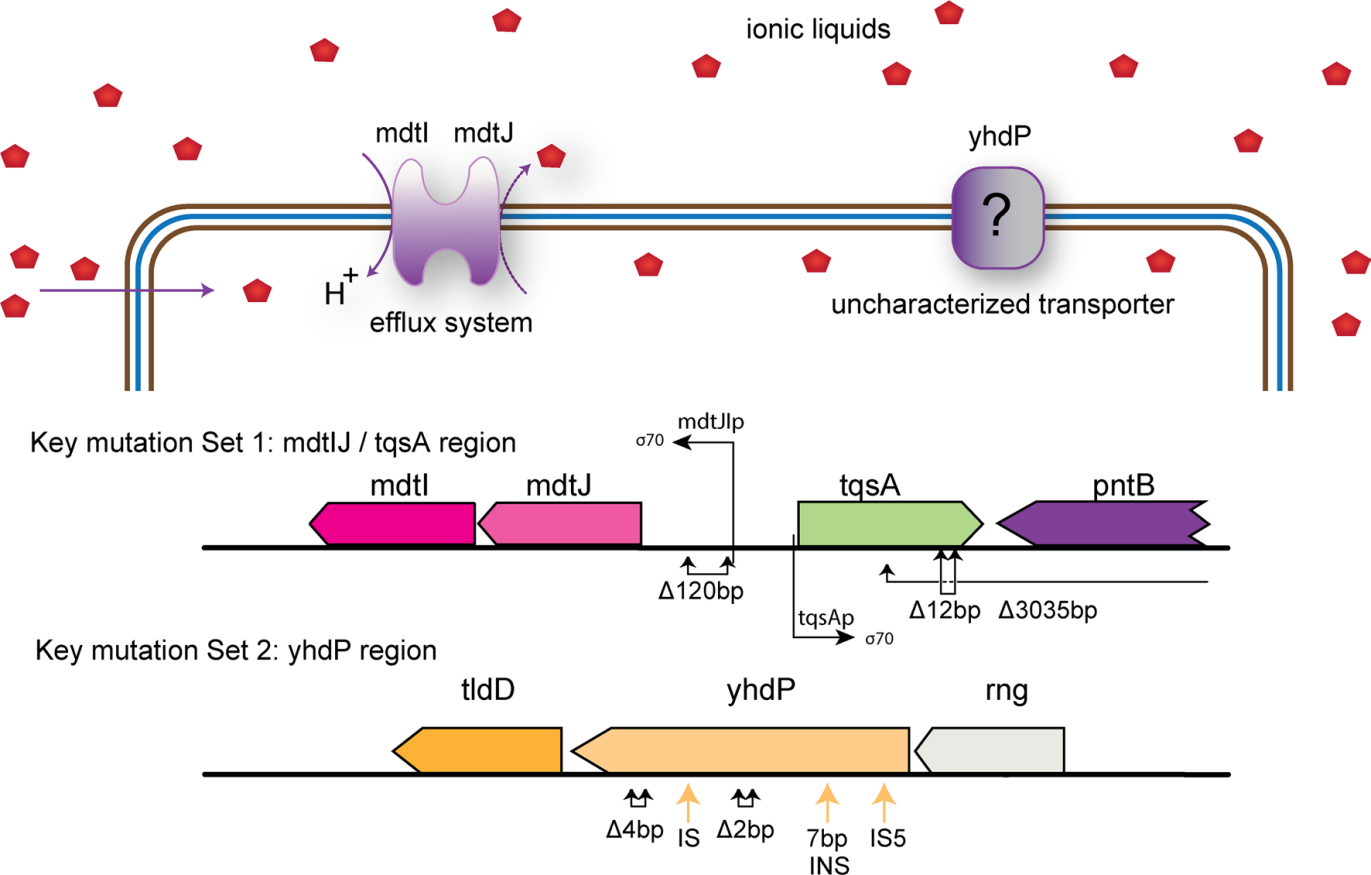
A diagram of the cell showing processes associated with key mutations. A cartoon diagram of key mutations in potential causal genetic regions identified in the evolved strains. A total of eight mutations are represented amongst different genetic regions. A Δ120 bp deletion was found in the non-coding region of *mdtJ*, near the promoter, and its neighboring gene *tqsA*. Two other structural changes were found in the *tqsA* gene—one was an intragenic in-frame Δ12 bp deletion, the other was a Δ3035 bp deletion which included a major section of *tqsA* and the *pntB* and *pntA* genes located next to *tqsA* on the chromosome. Finally, five structural changes were identified in *yhdP* gene. These were two out-of-frame short deletions (Δ2 bp and Δ4 bp), two intergenic IS mobile element insertions, and a short 7 bp insertion/duplication. These mutations indicate a probable loss-of-function of *yhdP*

The key mutations identified from this study provide insights into the potential mechanisms of tolerance phenotypes in the evolved strains. The most prevalent and shared mutations observed were in the *mdtJ*/*tqsA* intergenic region, as well as in the *tqsA* gene, and in the *yhdP* gene (Fig. 4). The key mutations identified in this study were specific to ILs when compared to a control experiment where K-12 MG1655 was evolved on M9 glucose minimal medium at the same temperature but without any stress from ILs [17]. Thus, it appears that modulating transport, likely of ILs, in and out of the cell is crucial for tolerance to the ILs tested here and likely similar compounds. This finding further supports the focus of rationally modulating transport systems in engineering tolerance [12, 23, 46]. However, identifying which transporters are critical for tolerance in a given strain de novo is difficult, therefore making the use of TALE a powerful approach.

A genetic-level analysis of the specific multidrug transport system mutation observed in both strains provides a glimpse into the mechanistic impact of these mutations. The most prevalent Δ120 bp deletion in the intergenic region between *mdtJ* and *tqsA* likely disrupts H-NS binding sites in the *mdtJI* promotor region, which is then believed to relieve negative repression of transcription of *mdtJI* by H-NS, given its role as a steric hindrance protein [47]. Supporting this, when H-NS is absent, a ninefold increase in *mdtJI* expression occurs as compared to wild type *E. coli*, and consequently the activity of MdtJI as well [47]. This finding further highlights the likely active role of this small multi-drug resistance (SMR) efflux pump in the resistance mechanism. Similar work has shown such pumps to be active on a wide range of inhibitory compounds [23, 46–48]. It is noteworthy that in the control experiment [17], an intergenic *hns*/*tdk* mutation was reported in almost all evolved endpoints where *hns* was determined to be upregulated and conferred a fitness advantage, i.e., fast growth rate, likely through subsequent downregulation of stress responses. Given that no similar intergenic *hns*/*tdk* mutations were seen in this work with ILs present during the evolution, this finding further supports the importance of high expression of *mdtJI* towards tolerance of the ILs examined here and the benefit of control ALE experiments.

The other key mutated region, in this case one gene, identified in the tolerant clones was in *yhdP*. The *yhdP* gene encodes a predicted transporter [29]. The occurrence of five unique mutations, all interpreted to be loss of function mutations, imply that removing this gene is a viable strategy for increased IL tolerance. However, the specific mechanism is unclear as to what metabolite is pumped in or out of the cell to provide the increased fitness. One can speculate that ILs could enter through this transporter, but this has yet to be verified. Future work could include an effort to definitively assign the causality of key mutations. For example, expression profiling could be performed on isolates or reconstructed strains carrying only the Δ120 bp deletion in the intergenic region between *mdtJ* and *tqsA*. Such transcription levels could help focus on the impact of *mdtJ* and/or *tqsA* and lead to a better understanding of the underlying mechanisms of tolerance.

The TALE approach of independently passaging multiple populations in an automated, strictly-controlled platform, coupled with next-generation sequencing resulted in an effective process for generating tolerant strains and for revealing the key causal mutations. Sequencing the whole genome revealed mutational changes in the evolved strains when compared to the reference strains. However, relating a specific mutation or a set of mutations, i.e., genotype, to the apparent phenotype in certain conditions is time-consuming [39, 49, 50]. The use of multiple independent replicates allowed for the identification of mutations in the same gene or genetic region multiple times across different TALE experiments. This approach of using many replicates to decipher the causality of a mutation or set of mutations in a given strain appeared effective given that the best performing clones, MG4.7 and MG3.10, possessed such key shared mutations. To validate the key mutations identified, as well as to confirm the efficacy of the evolution process, the selected clones were compared to rationally-designed strains in two different media with closely similar ILs. The performance of TALE-derived strains was superior, which indicated the efficacy of utilizing TALE and pointed to the identified mutations in the generated the strains.

In summary, utilizing the TALE approach outlined here to generate IL-tolerant strains resulted in the generation of promising platform strains with enhanced tolerance toward high concentrations of ILs [up to 11.9% (w/v)]. The approach used to identify and interpret the key causal mutations using whole genome sequencing complemented with analyzing isolates from multiple independent populations, and multiple isolates from each population, was successful in revealing the key mutations involved in IL tolerance phenotypes. The most striking identified key mutations appeared to involve modulation of transport mechanisms, possibly the direct transport of ILs into and out of the cell. The results of this study and the approach used to generate tolerant strains can be expanded to other conditions, strains, and selection criteria, which would help in fast-tracking the utilization of alternative renewable feedstocks, as well as to better understand tolerance mechanisms for inhibitory compounds.

## Additional files

**Additional file 1.** All of the whole genome sequencing results.

**Additional file 2.** Additional text, tables and figures.

## Abbreviations

IL: ionic liquid
TALE: tolerance adaptive laboratory evolution
ALE: adaptive laboratory evolution
*E. coli*: *Escherichia coli*
SMR: small multi-drug resistance

## References

[1] Johnson JM, Coleman MD, Gesch R, Jaradat A, Mitchell R, Reicosky D, Wilhelm WW (2007). Biomass, bioenergy crops in the United States: a changing paradigm. Am J Plant Sci Biotechnol.

[2] Hoffert MI, Caldeira K, Benford G, Criswell DR, Green C, Herzog H (2002). Advanced technology paths to global climate stability: energy for a greenhouse planet. Science.

[3] Moe ST, Janga KK, Hertzberg T, Hägg MB, Øyaas K, Dyrset N (2012). Saccharification of lignocellulosic biomass for biofuel and biorefinery applications A renaissance for the concentrated acid hydrolysis?. Energy Procedia..

[4] Jönsson LJ, Martín C (2016). Pretreatment of lignocellulose: formation of inhibitory by-products and strategies for minimizing their effects. Bioresour Technol.

[5] Sun N, Liu H, Sathitsuksanoh N, Stavila V, Sawant M, Bonito A, et al. Production and extraction of sugars from switchgrass hydrolyzed in ionic liquids. Biotechnol Biofuels. 2013;6:39. http://biotechnologyforbiofuels.biomedcentral.com/articles/10.1186/1754-6834-6-39. Accessed 13 Oct 2017. 10.1186/1754-6834-6-39 PMC3621597 23514699

[6] Li C, Tanjore D, He W, Wong J, Gardner JL, Sale KL (2013). Scale-up and evaluation of high solid ionic liquid pretreatment and enzymatic hydrolysis of switchgrass. Biotechnol Biofuels..

[7] Li C, Knierim B, Manisseri C, Arora R, Scheller HV, Auer M (2010). Comparison of dilute acid and ionic liquid pretreatment of switchgrass: biomass recalcitrance, delignification and enzymatic saccharification. Bioresour Technol..

[8] Ruegg TL, Kim E-M, Simmons BA, Keasling JD, Singer SW, Lee TS (2014). An auto-inducible mechanism for ionic liquid resistance in microbial biofuel production. Nat Commun..

[9] Frederix M, Hütter K, Leu J, Batth TS, Turner WJ, Rüegg TL (2014). Development of a native *Escherichia coli* induction system for ionic liquid tolerance. PLoS ONE.

[10] Reddy AP, Simmons CW, Claypool J, Jabusch L, Burd H, Hadi MZ (2012). Thermophilic enrichment of microbial communities in the presence of the ionic liquid 1-ethyl-3-methylimidazolium acetate. J Appl Microbiol.

[11] Qin D, Hu Y, Cheng J, Wang N, Li S, Wang D (2016). An auto-inducible *Escherichia coli* strain obtained by adaptive laboratory evolution for fatty acid synthesis from ionic liquid-treated bamboo hydrolysate. Bioresour Technol..

[12] Frederix M, Mingardon F, Hu M, Sun N, Pray T, Singh S (2016). Development of an *E. coli* strain for one-pot biofuel production from ionic liquid pretreated cellulose and switchgrass. Green Chem..

[13] LaCroix RA, Palsson BO, Feist AM (2017). A model for designing adaptive laboratory evolution experiments. Appl Environ Microbiol..

[14] Deatherage DE, Barrick JE (2014). Identification of mutations in laboratory-evolved microbes from next-generation sequencing data using breseq. Methods Mol Biol..

[15] Baeshen MN, Al-Hejin AM, Bora RS, Ahmed MMM, Ramadan HAI, Saini KS (2015). Production of biopharmaceuticals in *E. Coli*: current scenario and future perspectives. J Microbiol Biotechnol.

[16] Monk JM, Koza A, Campodonico MA, Machado D, Seoane JM, Palsson BO (2016). Multi-omics quantification of species variation of *Escherichia coli* links molecular features with strain phenotypes. Cell Syst..

[17] LaCroix RA, Sandberg TE, O’Brien EJ, Utrilla J, Ebrahim A, Guzman GI (2015). Use of adaptive laboratory evolution to discover key mutations enabling rapid growth of *Escherichia coli* K-12 MG1655 on glucose minimal medium. Appl Environ Microbiol..

[18] Lan W, Liu CF, Sun RC (2011). Fractionation of bagasse into cellulose, hemicelluloses, and lignin with ionic liquid treatment followed by alkaline extraction. J Agric Food Chem..

[19] Lee SH, Doherty TV, Linhardt RJ, Dordick JS (2009). Ionic liquid-mediated selective extraction of lignin from wood leading to enhanced enzymatic cellulose hydrolysis. Biotechnol Bioeng..

[20] Lee DH, Feist AM, Barrett CL, Palsson B (2011). Cumulative number of cell divisions as a meaningful timescale for adaptive laboratory evolution of *Escherichia coli*. PLoS ONE..

[21] Lin JJ, Sancar A (1989). A new mechanism for repairing oxidative damage to DNA: (A)BC excinuclease removes AP sites and thymine glycols from DNA. Biochemistry..

[22] Horst JP, Wu TH, Marinus MG (1999). *Escherichia coli* mutator genes. Trends Microbiol.

[23] Minty JJ, Lesnefsky AA, Lin F, Chen Y, Zaroff TA, Veloso AB, et al. Evolution combined with genomic study elucidates genetic bases of isobutanol tolerance in *Escherichia coli*. Microb Cell Fact. 2011;10:18. http://microbialcellfactories.biomedcentral.com/articles/10.1186/1475-2859-10-18. Accessed 22 Sept 2016. 10.1186/1475-2859-10-18 PMC3071312 21435272

[24] Higashi K, Ishigure H, Demizu R, Uemura T, Nishino K, Yamaguchi A (2008). Identification of a spermidine excretion protein complex (MdtJI) in *Escherichia coli*. J Bacteriol.

[25] Clarke DM, Loo TW, Gillam S, Bragg PD (1986). Nucleotide sequence of the pntA and pntB genes encoding the pyridine nucleotide transhydrogenase of *Escherichia coli*. Eur J Biochem..

[26] Haverkorn van Rijsewijk BRB, Kochanowski K, Heinemann M, Sauer U (2016). Distinct transcriptional regulation of the two *Escherichia coli* transhydrogenases PntAB and UdhA. Microbiology.

[27] Sauer U, Canonaco F, Heri S, Perrenoud A, Fischer E (2004). The soluble and membrane-bound transhydrogenases UdhA and PntAB have divergent functions in NADPH metabolism of *Escherichia coli*. J Biol Chem.

[28] Herzberg M, Kaye IK, Peti W, Wood TK (2006). YdgG (TqsA) controls biofilm formation in *Escherichia coli* K-12 through autoinducer 2 transport. J Bacteriol..

[29] Köstner M, Schmidt B, Bertram R, Hillen W (2006). Generating tetracycline-inducible auxotrophy in *Escherichia coli* and *Salmonella enterica* serovar typhimurium by using an insertion element and a hyperactive transposase. Appl Environ Microbiol.

[30] Conrad TM, Frazier M, Joyce AR, Cho B-K, Knight EM, Lewis NE (2010). RNA polymerase mutants found through adaptive evolution reprogram *Escherichia coli* for optimal growth in minimal media. Proc Natl Acad Sci USA.

[31] Nedea EC, Markov D, Naryshkina T, Severinov K (1999). Localization of *Escherichia coli* rpoC mutations that affect RNA polymerase assembly and activity at high temperature. J Bacteriol..

[32] Sandberg TE, Pedersen M, Lacroix RA, Ebrahim A, Bonde M, Herrgard MJ (2014). Evolution of *Escherichia coli* to 42 °C and subsequent genetic engineering reveals adaptive mechanisms and novel mutations. Mol Biol Evol.

[33] Phadtare S, Inouye M (2001). Role of CspC and CspE in regulation of expression of RpoS and UspA, the stress response proteins in *Escherichia coli*. J Bacteriol..

[34] Bae W, Xia B, Inouye M, Severinov K (2000). *Escherichia coli* CspA-family RNA chaperones are transcription antiterminators. Proc Natl Acad Sci USA.

[35] Wu X, Altman R, Eiteman MA, Altman E (2014). Adaptation of *Escherichia coli* to elevated sodium concentrations increases cation tolerance and enables greater lactic acid production. Appl Environ Microbiol..

[36] Kelly KO, Deutscher MP (1992). Characterization of *Escherichia coli* RNase PH. J Biol Chem..

[37] Poulsen P, Bonekamp F, Jensen KF (1984). Structure of the *Escherichia coli* pyrE operon and control of pyrE expression by a UTP modulated intercistronic attentuation. Embo J..

[38] Jensen KF (1993). The *Escherichia coli* K-12 “wild types” W3110 and MG1655 have an rph frameshift mutation that leads to pyrimidine starvation due to low pyrE expression levels. J Bacteriol..

[39] Conrad TM, Joyce AR, Applebee MK, Barrett CL, Xie B, Gao Y, et al. Whole-genome resequencing of *Escherichia coli* K-12 MG1655 undergoing short-term laboratory evolution in lactate minimal media reveals flexible selection of adaptive mutations. Genome Biol. 2009;10:R118. http://scholarscompass.vcu.edu/cmsc_pubs. Accessed 17 Oct 2016. 10.1186/gb-2009-10-10-r118 PMC2784333 19849850

[40] Roberts JW (1969). Termination factor for RNA synthesis. Nature..

[41] Goodarzi H, Bennett BD, Amini S, Reaves ML, Hottes AK, Rabinowitz JD (2010). Regulatory and metabolic rewiring during laboratory evolution of ethanol tolerance in *E. coli*. Mol Syst Biol..

[42] Haft RJF, Keating DH, Schwaegler T, Schwalbach MS, Vinokur J, Tremaine M (2014). Correcting direct effects of ethanol on translation and transcription machinery confers ethanol tolerance in bacteria. Proc Natl Acad Sci..

[43] Isik M, Sardon H, Mecerreyes D (2014). Ionic liquids and cellulose: dissolution, chemical modification and preparation of new cellulosic materials. Int J Mol Sci.

[44] He B, Smith JM, Zalkin H (1992). *Escherichia coli* purB gene: cloning, nucleotide sequence, and regulation by purR. J Bacteriol..

[45] Yu C, Simmons BA, Singer SW, Thelen MP, VanderGheynst JS (2016). Ionic liquid-tolerant microorganisms and microbial communities for lignocellulose conversion to bioproducts. Appl Microbiol Biotechnol..

[46] Bay DC, Stremick CA, Slipski CJ, Turner RJ (2017). Secondary multidrug efflux pump mutants alter *Escherichia coli* biofilm growth in the presence of cationic antimicrobial compounds. Res Microbiol..

[47] Leuzzi A, Di Martino ML, Campilongo R, Falconi M, Barbagallo M, Marcocci L (2015). Multifactor regulation of the MdtJI polyamine transporter in *Shigella*. PLoS ONE.

[48] Yung PY, Lo Grasso L, Mohidin AF, Acerbi E, Hinks J, Seviour T (2016). Global transcriptomic responses of *Escherichia coli* K-12 to volatile organic compounds. Sci Rep..

[49] Herring CD, Glasner JD, Blattner FR (2003). Gene replacement without selection: regulated suppression of amber mutations in *Escherichia coli*. Gene.

[50] Utrilla J, O’Brien EJ, Chen K, McCloskey D, Cheung J, Wang H (2016). Global rebalancing of cellular resources by pleiotropic point mutations illustrates a multi-scale mechanism of adaptive evolution. Cell Syst..

