# Supplementary material for: Generation of a platform strain for ionic liquid tolerance using adaptive laboratory evolution
Source: Microb Cell Fact. 2017 Nov 16;16:204. doi: 10.1186/s12934-017-0819-1 (PMC5691611; doi:10.1186/s12934-017-0819-1)
Supplement: Supplementary file 2 — Additional file 2. Additional text, tables and figures. [file 12934_2017_819_MOESM2_ESM.docx]

Additional Text

Table of Contents

[Additional Text 2](#_Toc490736369)

[Figures 5](#_Toc490736370)

[Figure S1 8](#_Toc490736371)

[Figure S2 7](#_Toc490736372)

[Figure S3 5](#_Toc490736373)

[Tables 10](#_Toc490736374)

[Table S1 10](#_Toc490736375)

[Table S2 12](#_Toc490736376)

[Table S3 10](#_Toc490736377)

[Table S4 11](#_Toc490736378)

[Table S5 13](#_Toc490736379)

[Table S6 14](#_Toc490736380)

# Additional Text

Detailed Analysis of Secondary Screening Results

Whole genome sequencing was performed on clonal isolates from TALE experiments to increasing IL concentrations and they were clustered based on their genotype into three clusters:

- 1. **Genetically-identical clones**: It was found that for each of the strain and IL combination, there were clones that shared identical genotypes. Thus, as a measure of validation, the identical genotypic clones’ phenotypic properties were compared and it was found that there were different levels of agreement.
  2. **Clustering of Genetically-similar clones:** There were several mutated genotypes that clustered together based on shared mutations (an expected outcome as multiple clones were isolated from the same population). This clustering was roughly based on the appearance of a large number of shared identical mutations (n>= 5) or clones sharing a large number of identical mutations overall (m>=70%). Overall, there were 2, 3, 2, and 3 genetically-similar clusters for the MG1655/([C_4_C_1_Im]CL), DH1/([C_4_C_1_Im]CL), MG1655/[C_2_C_1_Im][OAC], and DH1/[C_2_C_1_Im][OAC] conditions, respectively.
  3. **Hypermutating Clones:** There were three hyper-mutating clones (one MG1655 and two DH1 strains) isolated during the analysis (strains that had and average number of mutations of 268 for a mutant from and with 39±4 for hyper-mutating from DH1, as compared to 6±1 for non-hyper-mutating clones).

All of the phenotypic properties of the mutations identified for the clustered genotypes are given in **Table S2** and **Table S3** for MG1655 and DH1 respectively. The method used to choose representative clones from each genetically similar ‘Set’ is summarized in **Figure S2**. These representative clones were used to screen performance for enhanced performance.

1. *E. coli* MG1655/ ([C_4_C_1_Im]CL)

*E. coli* MG1655/ ([C_4_C_1_Im]CL) according to **Table S2** has 3 ‘sets’, two of the sets are not shown as they did not grow (**Table S5**). These sets will be excluded from the analysis herein. Set MG-BM-3A has 2 genetically-identical clones with %RSD of 0% and 2% of average growth rate and final OD, respectively. Set MG-BM-3(B-E) is genetically-similar with a high level of similarity based on phenotypic data with overall of %RSD of 9%, 5% for average growth rate and final optical density, respectively. Thus, overall, the identical and similar sets had a very similar performance in the secondary screen.

1. *E. coli* MG1655/ [C_2_C_1_Im][OAC]

*E. coli* MG1655/[C_2_C_1_Im][OAC] has two closely similar genotypes ‘sets’, the base set MG-EM-1 and MG-EM-1A with additional mutations over that of MG-EM-1A. The first set, base set MG-EM-1, clones that are sharing the same basic mutated genes, has varying growth and final optical density. With overall of %RSD of 40% and 52% of average growth rate and final optical density, respectively. The second set, MG-EM-1A, has clones that were genetically-identical with %RSD of 9% and 34% of average growth rate and final OD, respectively. Clones from set MG-EM-1A have the same mutation as in MG-EM-1, plus one additional mutation. The variability of MG-EM-1A clones (n=9 clones in the set) suggests more mutations in these clones than those which were detected (this currently under further investigation).

1. *E. coli* DH1/ ([C_4_C_1_Im]CL)

This combination has 4 genetically-similar sets; DH-BM-1, DH-BM-1A, DH-BM-2 and DH-BM-3(A-B). Set DH-BM-1 and set DH-BM-1A, are closely genetically identical expect in a single mutated gene in set DH-BM-1A. Although set DH-BM-1 has four genetically-identical clones, it has a largely variable phenotypic data with overall %RSD of 55% and 24% for average growth rate and final optical density, respectively. Set DH-BM-3(A-B) has genetically-similar clones sharing nearly identical phenotypic data with %RSD of 6% and 0% for average growth rate and final OD, respectively. DH-BM-2 has clones with genetically-identical genotypes, but possesses varying phenotypic data but still comparatively close with overall all %RSD of 24% and 20% for average growth rate and final optical density.

1. *E. coli* DH1/ [C_2_C_1_Im][OAC]

These evolved clones are clustered into 5 genotype sets; DH-EM-1, DH-EM-2A, DH-EM-2(B-C), DH-EM-3A and DH-EM-3(B-D). Set DH-EM-2A and DH-EM-2(B-C) are relatively genetically-similar. Clones from set DH-EM-2A, are identical with phenotypic agreement with overall %RSD of 0% and 63% for average growth rate and final optical density, respectively. Set DH-EM-2(B-C) has relatively genetically-similar with 4 mutated genes different but the phenotypic data has high level of agreement with overall %RSD of 10% and 15% for average growth rate and final optical density, respectively. Both sets DH-EM-3A and set DH-EM-3(B-D) are fairly genetically-identical except with three mutated genes. Where set ‘DH-EM-3(B-D)’ has a little variable phenotypic data with overall of %RSD of 10% and 15%, set ‘DH-EM-3A’ has closely identical data with 0% and 0% for average growth rate and final optical density, respectively.

# Figures

**Figure S1**: The resulting growth curves from primary screen for 10 isolates from all evolved populations on ([C_4_C_1_Im]CL), panel A and [C_2_C_1_Im][OAC], panel B based on ILs concentration Table 1. Growth curves for each isolate were inspected for those exhibiting robust growth or unique growth profiles such as exhibiting reduced lag-times, increased final densities, and increased in the apparent growth rates. Candidates were selected for comparisons with rationally-designed strains and for cross-tolerance.


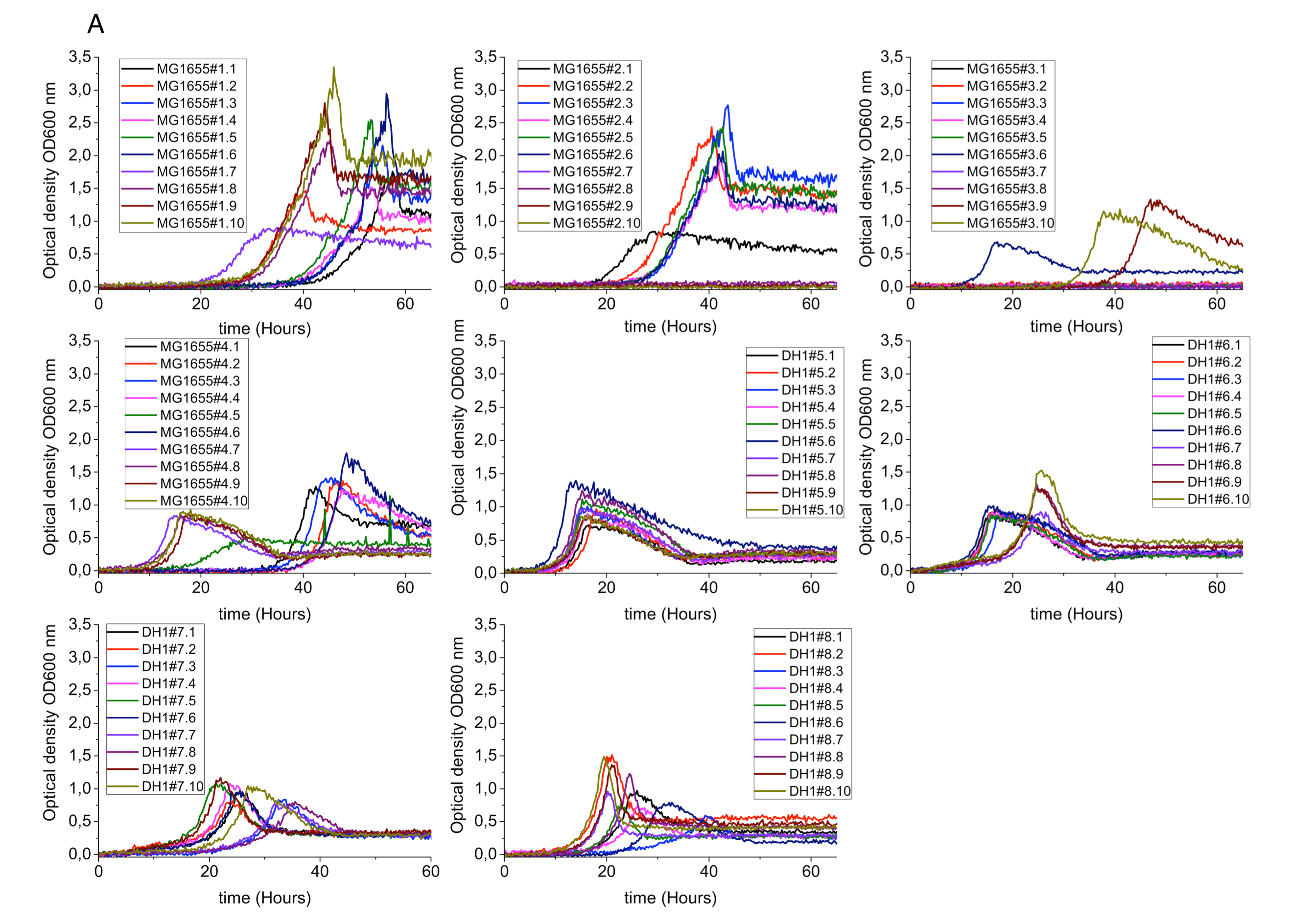


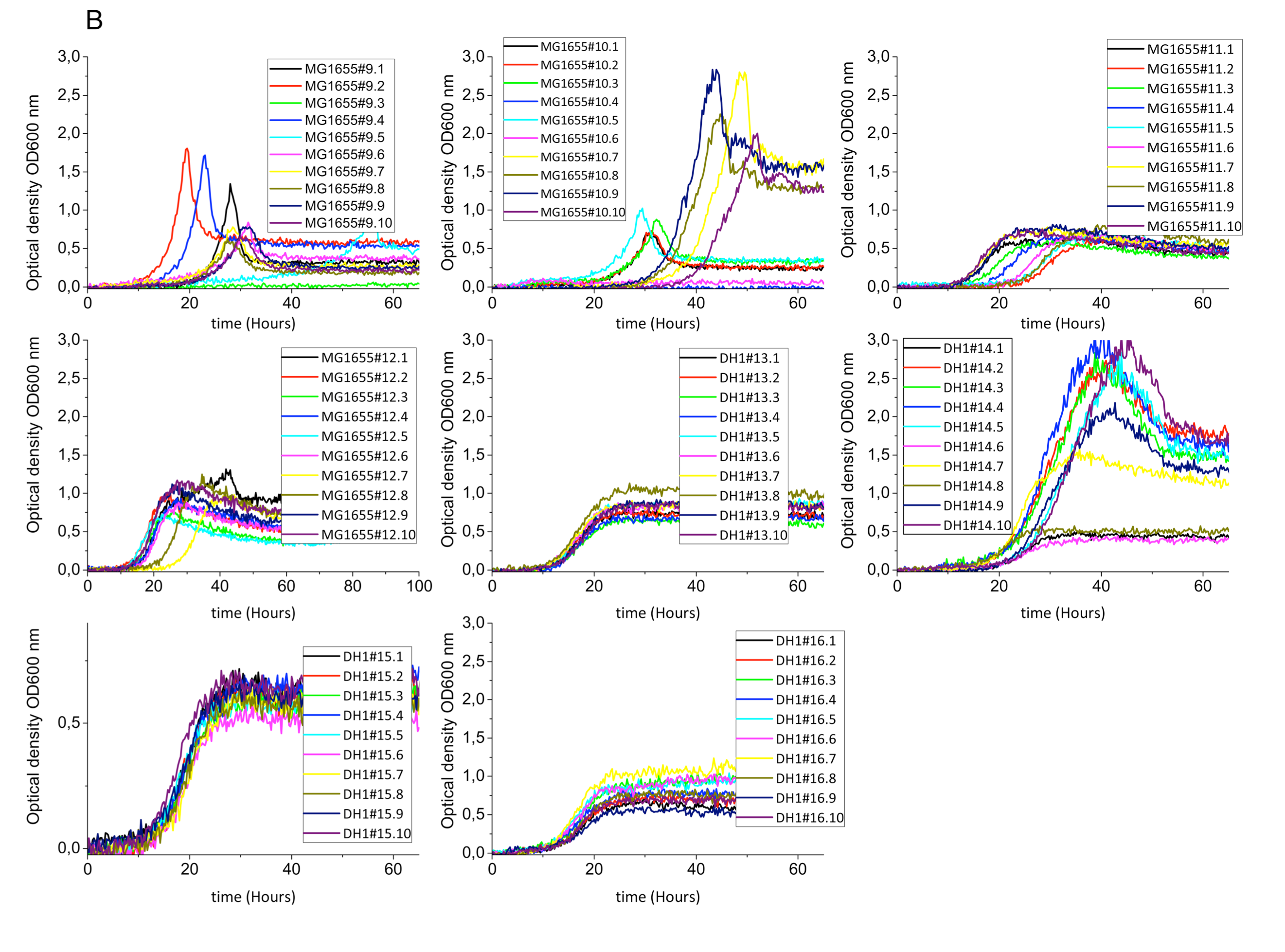


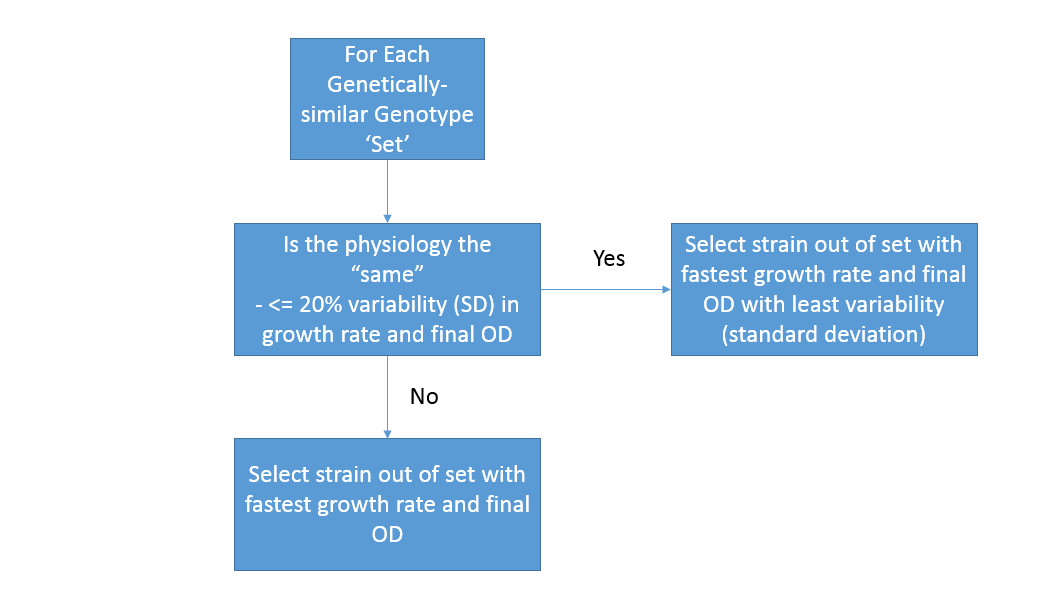


**Figure S2: Logic cart for selecting a representative clone from each genetically-similar set**. Such clones were subjected to further validation. For each genetically-similar cluster set, if the physiology is the same with a % RSD <=20% variability in growth rate and final OD, the selection was made based on the clone with the fastest growth rate and highest final OD with least variability. Alternatively, clones with the fastest growth rate and highest final OD were selected.

**Figure S3: Plots of populations growth rate trajectories versus the change in ILs concentration of fourteen of the TALE experiments**. (ALE#1-4 and ALE#9-12) E. coli K-12 MG1655 populations evolved on M9-glucose minimal medium with ([C_4_C_1_Im]CL) and [C_2_C_1_Im][OAC], respectively) and, (ALE#5-8 and ALE#13-16) E. coli DH1 populations on M9-glucose minimal medium with ([C_4_C_1_Im]CL) and [C_2_C_1_Im][OAC], respectively). Depicted are plots of fitness trajectories and IL concentration versus cumulative cell divisions (CCD) experienced by the cultures for fourteen of the total sixteen individual experiments. IL concentration was increasing in cycles of a step-wise routine when there was improve in each corresponding population’s fitness, i.e. growth rate.


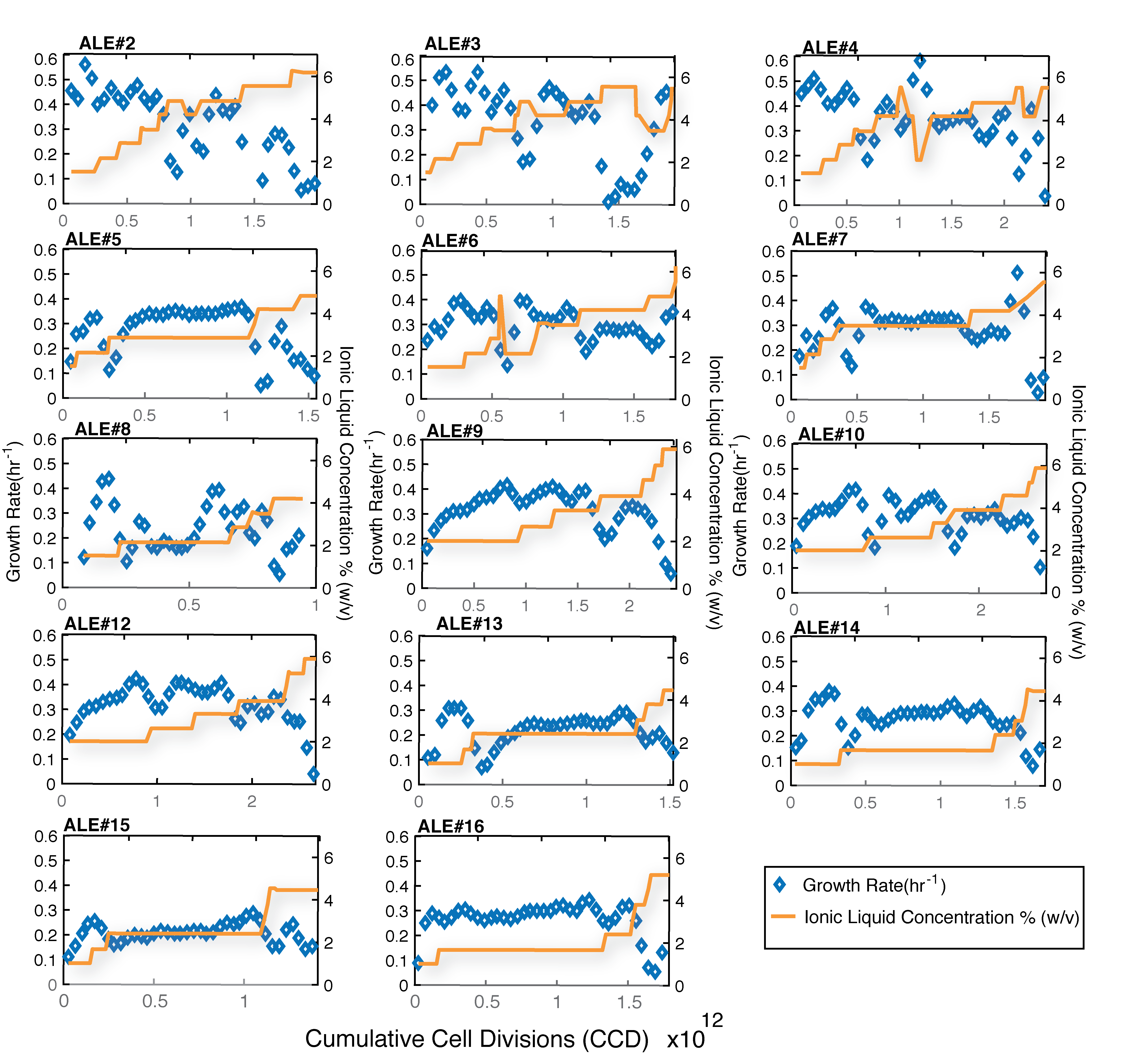


#

# Tables

**Table S1: Screening for tolerance in the wild type strains. Each of the two *Escherichia coli* (MG1655 and DH1) had two replicates in each different concentration**. Five different conditions of ILs concentration (1%, 1.5%, 2%, 2.5%, and 3%) have been used throughout the characterization experiment. The results from the initial screening were used to decide the start concentration for the main evolution on ILs. The data represented here is only for screens that showed growth in each specific concentration. The character # denotes the number of replicate in each characterization experiment. Samples were run replicates n=2 and standard deviations is given for final optical density and growth rates.

| IL type | *E. coli* strain | IL concentration %(w/v) | Final OD(OD_600nm_) | Growth Rate (hr^-1^) |
| --- | --- | --- | --- | --- |
| ([C_4_C_1_Im]CL) | MG1655 | 1 | 0.4±<0.01 | 0.32±<0.01 |
|  | MG1655 | 1.5 | 0.4±<0.01 | 0.29±<0.01 |
|  | DH1 | 1 | 0.3±<0.01 | 0.12±0.02 |
|  | DH1 | 1.5 | 0.3±<0.01 | 0.17±0.015 |
| [C2C1Im][OAC] | MG1655 | 1 | 0.5±<0.01 | 0.3±0.035 |
|  | MG1655 | 1.5 | 0.5±<0.01 | 0.2±<0.01 |
|  | MG1655 | 2 | 0.44±<0.01 | 0.11±<0.01 |
|  | MG1655 | 2.5 | 0.40±0.03 | 0.06±0.015 |
|  | MG1655 | 3 | 0.23±0.02 | 0.026±<0.01 |
|  | DH1 | 1 | 0.14±<0.01 | 0.09±<0.01 |
|  | DH1 | 1.5 | 0.08±<0.01 | 0.065±0.01 |

**Table S2: genotypic sets of evolved E. coli k-12 MG1655 clones in two different IL types and their phenotypic properties**.

| Strain name | IL type | IL Conc. | Genetically-similar cluster set | Average growth rate (hr-1) | STDEV (hr-1) | Ave-final OD600 | STDEV (hr-1) | Average LAG –TIME (HR) | STDEV (hr.) |
| --- | --- | --- | --- | --- | --- | --- | --- | --- | --- |
| **MG1655#2.3** | ([C_4_C_1_Im]CL) | 5.4% | MG-BM-3A | 0.25 | 0.044 | 0.90 | 0.094 | 5.03 | 0.13 |
| **MG1655#4.7** | ([C_4_C_1_Im]CL) | 5.4% | MG-BM-3A | 0.26 | 0.024 | 0.87 | 0.039 | 4.88 | 0.04 |
|  |  |  | **Average** | **0.25** |  | **0.88** |  | **4.95** |  |
|  |  |  | **STD** | **0.00** |  | **0.02** |  | **0.08** |  |
|  |  |  | **CV** | **0%** |  | **2%** |  | **2%** |  |
| **MG1655#3.6** | ([C_4_C_1_Im]CL) | 5.4% | MG-BM-3B | 0.26 | 0.007 | 0.82 | 0.027 | 14.88 | 0.04 |
| **MG1655#3.9** | ([C_4_C_1_Im]CL) | 5.4% | MG-BM-3C | 0.25 | 0.025 | 0.85 | 0.031 | 14.20 | 5.08 |
| **MG1655#3.10** | ([C_4_C_1_Im]CL) | 5.4% | MG-BM-3D | 0.31 | 0.009 | 0.94 | 0.040 | 10.85 | 1.24 |
| **MG1655#4.5** | ([C_4_C_1_Im]CL) | 5.4% | MG-BM-3E | 0.30 | 0.015 | 0.84 | 0.082 | 3.19 | 1.17 |
|  |  |  | **Average** | **0.28** |  | **0.86** |  | **10.78** |  |
|  |  |  | **STD** | **0.02** |  | **0.05** |  | **4.64** |  |
|  |  |  | **CV** | **9%** |  | **5%** |  | **43%** |  |
| **MG1655#9.2** | [C_2_C_1_Im][OAC] | 4.6% | MG-EM-1 | 0.12 | 0.013 | 0.07 | 0.007 | 0.13 | 0.011 |
| **MG1655#9.4** | [C_2_C_1_Im][OAC] | 4.6% | MG-EM-1 | 0.14 | 0.001 | 1.04 | 0.006 | 0.41 | 0.005 |
| **MG1655#9.8** | [C_2_C_1_Im][OAC] | 4.6% | MG-EM-1 | 0.05 | 0.003 | 0.12 | 0.002 | 0.17 | 0.01 |
| **MG1655#10.1** | [C_2_C_1_Im][OAC] | 4.6% | MG-EM-1 | 0.10 | 0.003 | 1.32 | 0.002 | 1.24 | 0.004 |
| **MG1655#11.1** | [C_2_C_1_Im][OAC] | 4.6% | MG-EM-1 | 0.17 | 0.012 | 1.44 | 0.001 | 0.98 | 0.006 |
| **MG1655#11.5** | [C_2_C_1_Im][OAC] | 4.6% | MG-EM-1 | 0.22 | 0.021 | 1.64 | 0.012 | 1.61 | 0.01 |
| **MG1655#11.10** | [C_2_C_1_Im][OAC] | 4.6% | MG-EM-1 | 0.23 | 0.003 | 1.69 | 0.004 | 1.55 | 0.006 |
| **MG1655#12.3** | [C_2_C_1_Im][OAC] | 4.6% | MG-EM-1 | 0.16 | 0.025 | 1.24 | 0.024 | 1.42 | 0.02 |
| **MG1655#12.7** | [C_2_C_1_Im][OAC] | 4.6% | MG-EM-1 | 0.27 | 0.005 | 1.1 | 0.03 | 1.9 | 0.015 |
|  |  |  | **Average** | **0.16** |  | **1.07** |  | **1.04** |  |
|  |  |  | **STD** | **0.065** |  | **0.56** |  | **0.62** |  |
|  |  |  | **CV** | **40%** |  | **52%** |  | **60%** |  |
| **MG1655#10.8** | [C_2_C_1_Im][OAC] | 4.6% | MG-EM-1A | 0.11 | 0.021 | 1.52 | 0.012 | 1.36 | 0.009 |
| **MG1655#10.9** | [C_2_C_1_Im][OAC] | 4.6% | MG-EM-1A | 0.09 | 0.003 | 0.74 | 0.004 | 1.99 | 0.006 |
|  |  |  | **Average** | **0.10** |  | **1.13** |  | 1.67 |  |
|  |  |  | **STD** | **0.01** |  | **0.39** |  | 0.32 |  |
|  |  |  | **CV** | **9%** |  | **34%** |  | 19% |  |

**Table S3: genotypic sets of evolved E. coli DH1 clones in two different ILs and their phenotypic properties**.

| Strain name | IL type | IL Conc. | Genetically-similar cluster set | Average growth rate (hr-1) | STDEV (hr-1) | Ave-final OD600 | STDEV (hr-1) | AVerage LAG –TIME (HR) | STDEV (hr) |
| --- | --- | --- | --- | --- | --- | --- | --- | --- | --- |
| DH1#5.3 | [C_4_C_1_Im]CL | 4.6% | DH-BM-3A | 0.32 | 0.005 | 0.96 | 0.012 | 12.51 | 0.075 |
| DH1#6.1 | [C_4_C_1_Im]CL | 4.6% | DH-BM-3B | 0.29 | 0.01 | 0.96 | 0.009 | 14.85 | 0.044 |
|  |  |  | **Average** | **0.30** |  | **0.96** |  | 13.68 |  |
|  |  |  | **STD** | **0.02** |  | **0.00** |  | 1.17 |  |
|  |  |  | **CV** | **6%** |  | **0%** |  | 9% |  |
| DH1#5.7 | [C_4_C_1_Im]CL | 4.6% | DH-BM-2 | 0.35 | 0.026 | 0.41 | 0.657 | 13.90 | 0.572 |
| DH1#5.10 | [C_4_C_1_Im]CL | 4.6% | DH-BM-2 | 0.58 | 0.023 | 0.61 | 0.122 | 8.80 | 1.059 |
|  |  |  | **Average** | **0.47** |  | **0.51** |  | **11.35** |  |
|  |  |  | **STD** | **0.11** |  | **0.10** |  | **2.55** |  |
|  |  |  | **CV** | **24%** |  | **20%** |  | **22%** |  |
| DH1#6.7 | [C_4_C_1_Im]CL | 4.6% | DH-BM-1 | 0.36 | 0.033 | 0.99 | 0.094 | 5.62 | 0.154 |
| DH1#6.8 | [C_4_C_1_Im]CL | 4.6% | DH-BM-1 | 0.07 | 0.012 | 0.83 | 0.031 | 10.69 | 0.087 |
| DH1#7.2 | [C_4_C_1_Im]CL | 4.6% | DH-BM-1 | 0.14 | 0.043 | 0.78 | 0.043 | 20.02 | 1.023 |
| DH1#7.5 | [C_4_C_1_Im]CL | 4.6% | DH-BM-1 | 0.25 | 0.025 | 1.39 | 0.014 | 11.49 | 3.604 |
|  |  |  | **Average** | **0.20** |  | **1.00** |  | **11.95** |  |
|  |  |  | **STD** | **0.11** |  | **0.24** |  | **5.17** |  |
|  |  |  | **CV** | **55%** |  | **24%** |  | **43%** |  |
| DH1#7.7 | [C_4_C_1_Im]CL | 4.6% | DH-BM-1A | 0.10 | 0.018 | 0.49 | 0.015 | 0.17 | 0.000 |
| DH1#13.2 | [C_2_C_1_Im][OAC] | 4.2% | DH-EM-2A | 0.15 | 0.021 | 2.30 | 0.090 | 10.00 | 0.000 |
| DH1#13.10 | [C_2_C_1_Im][OAC] | 4.2% | DH-EM-2A | 0.14 | 0.013 | 0.52 | 0.385 | 9.59 | 0.666 |
|  |  |  | **Average** | **0.15** |  | **1.41** |  | **9.79** |  |
|  |  |  | **STD** | **0.00** |  | **0.89** |  | **0.21** |  |
|  |  |  | **CV** | **0%** |  | **63%** |  | **2%** |  |
| DH1#14.2 | [C_2_C_1_Im][OAC] | 4.2% | DH-EM-2B | 0.20 | 0.027 | 0.36 | 0.304 | 4.91 | 1.083 |
| DH1#14.7 | [C_2_C_1_Im][OAC] | 4.2% | DH-EM-2C | 0.17 | 0.007 | 0.48 | 0.133 | 5.65 | 2.755 |
|  |  |  | **Average** | **0.18** |  | **0.42** |  | **5.28** |  |
|  |  |  | **STD** | **0.02** |  | **0.06** |  | **0.37** |  |
|  |  |  | **CV** | **10%** |  | **15%** |  | **7%** |  |
| DH1#13.8 | [C_2_C_1_Im][OAC] | 4.2% | DH-EM-1 | 0.23 | 0.007 | 0.60 | 0.085 | 8.57 | 3.393 |
| DH1#14.4 | [C_2_C_1_Im][OAC] | 4.2% | DH-EM-3B | 0.26 | 0.022 | 0.19 | 0.153 | 4.26 | 0.190 |
| DH1#15.2 | [C_2_C_1_Im][OAC] | 4.2% | DH-EM-3C | 0.28 | 0.057 | 0.57 | 0.148 | 6.33 | 0.259 |
| DH1#15.8 | [C_2_C_1_Im][OAC] | 4.2% | DH-EM-3D | 0.12 | 0.011 | 0.68 | 0.002 | 3.78 | 0.053 |
|  |  |  | **Average** | **0.22** |  | **0.48** |  | **4.79** |  |
|  |  |  | **STD** | **0.07** |  | **0.21** |  | **1.11** |  |
|  |  |  | **CV** | **31%** |  | **43%** |  | **23%** |  |
| DH1#16.6 | [C_2_C_1_Im][OAC] | 4.2% | DH-EM-3A | 0.30 | 0.048 | 0.26 | 0.004 | 3.53 | 0.106 |
| DH1#16.7 | [C_2_C_1_Im][OAC] | 4.2% | DH-EM-3A | 0.31 | 0.014 | 0.26 | 0.172 | 5.34 | 0.440 |
|  |  |  | **Average** | **0.30** |  | **0.26** |  | **4.43** |  |
|  |  |  | **STD** | **0.00** |  | **0.00** |  | **0.91** |  |
|  |  |  | **CV** | **0%** |  | **0%** |  | **20%** |  |

**Table S4: Properties of the TALE experiment. Cumulative cell divisions and total doublings underwent during the course of evolution for each corresponding TALE experiment**.

| Experiment Identifier (TALE) | Total CCD | Total Doublings |
| --- | --- | --- |
| 1 | 2.20x10^12^ | 371.20 |
| 2 | 1.20x10^12^ | 412.24 |
| 3 | 1.90x10^12^ | 384.76 |
| 4 | 2.42x10^12^ | 500.55 |
| 5 | 1.55x10^12^ | 342.30 |
| 6 | 1.80x10^12^ | 423.54 |
| 7 | 1.93x10^12^ | 417.50 |
| 8 | 0.95x10^12^ | 256.60 |
| 9 | 2.50x10^12^ | 503.00 |
| 10 | 2.72x10^12^ | 501.20 |
| 11 | 2.98x10^12^ | 551.90 |
| 12 | 2.70x10^12^ | 515.20 |
| 13 | 1.52x10^12^ | 435.80 |
| 14 | 1.70x10^12^ | 488.50 |
| 15 | 1.41x10^12^ | 409.90 |
| 16 | 1.79x10^12^ | 486.60 |

**Table S5** – List of clones that did not grow in the secondary screening - eliminated from the screening analysis

| Strain | IL-type | Screening IL concentration | Genetically-similar cluster set | Exclusion reason |
| --- | --- | --- | --- | --- |
| **MG1655#1.1** | ([C_4_C_1_Im]CL) | 5.4% | MG-BM-1 | NG |
| **MG1655#1.2** | ([C_4_C_1_Im]CL) | 5.4% | MG-BM-2A | NG |
| **MG1655#1.8** | ([C_4_C_1_Im]CL) | 5.4% | MG-BM-2B | NG |
| **MG1655#2.1** | ([C_4_C_1_Im]CL) | 5.4% | MG-BM-2C | NG |
| **MG1655#2.2** | ([C_4_C_1_Im]CL) | 5.4% | MG-BM-2 | NG |
| **MG1655#4.10** | ([C_4_C_1_Im]CL) | 5.4% | Hyper-mutating | - |
| **MG1655#12.8** | [C_2_C_1_Im][OAC] | 5.4% | MG-EM-1B | NG |
| **DH1#15.10** | [C_2_C_1_Im][OAC] | 4.2% | Hyper-mutating | - |
| **DH1#16.1** | [C_2_C_1_Im][OAC] | 4.2% | Hyper-mutating | - |

NG stands for no growth was detected on secondary screening

| strain | Gene | Mutation | Mutation | function | IL observed | Count |
| --- | --- | --- | --- | --- | --- | --- |
| MG1655 | rrfE | noncoding (12/120 nt) A→C | SNP | 5S ribosomal RNA of rrnE operon | 1 | 6 |
|  | rpsA | V122L (GTT→CTT) | SNP | 30S ribosomal subunit protein S1 | 1 | 4 |
|  | hyfG | E541K (GAG→AAG) | SNP | hydrogenase 4 subunit | 1 | 6 |
|  | exuR/yqjA | intergenic (+59/‑286) (+A) | INS | transcription repressor/Transporter | 1 | 6 |
|  | yobF | coding (141‑144/144 nt) IS5 (–) +4 bp | MOB | stress protein | 1 | 4 |
|  | yhcG | Q130* (CAG→TAG) | SNP | conserved protein |  | 4 |
|  | clsA | coding (902/1461 nt) Δ1 bp | DEL | cardiolipin synthase | 1 | 6 |
| DH1 | ECDH1_RS22450 /trxA | intergenic (‑1/+137) repeat_region (+) +4 bp :: Δ7 bp | MOB | electron transfer protein | 2 | 5 |
|  | essQ | coding (157‑161/216 nt) Δ3 bp :: repeat_region (+) +5 bp :: Δ1 bp | MOB | predicted S lysis protein | 2 | 5 |
|  | codB/prpE | intergenic (‑192/+45) Δ94 bp | DEL | cystosin transporter/propionyl-CoA synthetase | 2 | 2 |
|  | purR | E180G (GAA→GGA) | SNP | transcriptional repressor | 2 | 5 |
|  | ubiB | coding (557‑568/1641 nt) Δ12 bp | DEL | biosynthesis of ubiquinone-8 | 1 | 2 |
|  | yhjG | D189D (GAT→GAC) | SNP | membrane protein | 2 | 5 |

**Table S6:** Additional mutations found in each strain for the re-sequenced isolates. Shown are the key mutations in the same gene across different TALE experiments. Listed is a set of strain-specific genes for MG1655 and DH1 strains which were mutated only for a single time in different clones for each strain. The IL observed for each mutation is given as 1 and 2 for each of ([C_4_C_1_Im]CL) and [C_2_C_1_Im][OAC], respectively.
